# Supplementary material for: A DeepSeek-powered AI system for automated chest radiograph interpretation in clinical practice
Source: Nat Commun. 2026 May 7;17:6141. doi: 10.1038/s41467-026-72680-6 (PMC13365505; doi:10.1038/s41467-026-72680-6)
Supplement: Supplementary file 1 — Supplementary information [file 41467_2026_72680_MOESM1_ESM.pdf]

Contents

Supplementary Note 1 Model establishment. 2
Supplementary Note 2 MIMIC-CXR, CheXpert Plus and CXR-27 datasets. 4
Supplementary Note 3 Prospective medical centers. 5
Supplementary Note 4 Data cleaning of MIMIC-CXR and CheXpert Plus datasets. 6
Supplementary Note 5 Automated labeling tool for chest x-ray reports. 7
Supplementary Note 6 Multi-dimensional automated report generation metrics to evaluate report generation quality. 9
Supplementary Note 7 Standardized Training and Assessment for Subjective Evaluation 10
Supplementary Note 8 Radiology report quality scoring criteria (5-point likert scale). . 11
Supplementary Note 9 RADPEER scoring criteria 13
Supplementary Note 10 Sample size calculation 14
Supplementary Figure 1 Confusion matrix evaluation of large model-generated radiology reports. 15
Supplementary Figure 2 Receiver operating characteristic (ROC) curves (AUC<0.8) of 14 predictive features in the CXR-27 Test Set. 16
Supplementary Figure 3 Prospective Validation for Multi-Image Input Scenarios Based on 50 Patients with Posteroanterior and Lateral Chest X-rays. 17
Supplementary Figure 4 Interactive Interface for Professional Medical Imaging Analysis. 18
Supplementary Table 1 Baseline characteristics of the retrospective study. 19
Supplementary Table 2 Baseline characteristics of prospective study participants 20
Supplementary Table 3 Inter-rater reliability verification in the prospective study. 21
Supplementary Table 4 An example of AI improving junior radiologists' reporting accuracy. 22
Supplementary Table 5 Inter-rater reliability verification in the retrospective study 23
Supplementary Table 6 Automated report generation metrics. 24
Supplementary Table 7 Model performance evaluation using F1 scores on the CXR-27 test set. 25
Supplementary Table 8 An example of historical chest radiographs and current chest radiographs as model inputs. 26
Supplementary Table 9 An example of posteroanterior and lateral chest radiographs as model inputs. 27
Supplementary Table 10 An example of the model's suboptimal performance 28
References 29

Supplementary Note 1 Model establishment.

This study constructed a chest X-ray report generation system with collaborative large and small models based on Janus-Pro (1B) *(https://github.com/deepseek-ai/Janus)* and EVA-X *(https://github.com/hustvl/EVA-X)*. For Janus-Pro, the fine-tuning code refers to the open-source project LLaMA-Factory *(https://github.com/hiyouga/LLaMA-Factory)*, while the fine-tuning of EVA-X fully follows its code repository. In the final system, the small classification model first makes an initial disease classification diagnosis on the input image, and the large report generation model generates corresponding reports by integrating system prompts, user prompts (clinical information, disease classification diagnosis information, user input), and image input.

For the fine-tuning of Janus-Pro, all chest X-ray images are uniformly scaled such that their long sides reach a specified size, and the short sides are filled with a background color (RGB: 127, 127, 127), ultimately adjusted to 384×384 pixels. In addition, all imaging reports are organized into a format containing only "FINDINGS" or "FINDINGS" + "IMPRESSION".

The model underwent three stages of fine-tuning. The first stage focused on the report generation capability of the large model itself. The large model was initially fine-tuned based on the MIMIC-CXR dataset to enable it to master basic imaging interpretation and report generation skills. The second stage aimed at collaborative training of the large and small models. The large model was further fine-tuned based on the MIMIC-CXR and CheXpert Plus datasets to equip it with the ability to analyze clinical information, disease classification diagnosis information, comparative images (previous vs. current), and posteroanterior-lateral images. The third stage emphasized style transfer, where the model achieved alignment with the writing styles of multicenter reports based on the CXR-27 dataset.

All training was completed on 4 Nvidia A100 (40 GB) graphics cards, and some training parameters are shown as follows.

	Stage One	Stage Two	Stage Three

Learning rate	2e-4	2e-4	1e-4
LR scheduler	Constant	Constant	Constant
Weight decay	0.0	0.0	0.0
Gradient clip	1.0	1.0	1.0
Optimizer	AdamW(0.9,0.95)	AdamW(0.9,0.95)	AdamW(0.9,0.95)
Training steps	4800	3000	675
Batch size	256	256	128

All tests were completed on a single Nvidia GeForce RTX 3090 (24 GB) graphics card, and a comprehensive evaluation of the model's natural language generation (NLG) metrics and clinical metrics was conducted on the test set.

Supplementary Note 2 MIMIC-CXR, CheXpert Plus and CXR-27 datasets.

MIMIC-CXR (Medical Information Mart for Intensive Care - Chest X-Ray) is a large-scale publicly available medical imaging dataset containing over 370,000 chest X-ray images in DICOM format and 227,835 corresponding radiology reports in text format, covering imaging data from intensive care unit (ICU) patients[1]. MIMIC-CXR does not include patients' medical history information. Only posteroanterior chest radiographs were included in this study. The radiology reports, which are divided into "Findings" and "Impression" sections, have undergone de-identification to protect patient privacy. The dataset includes the following diagnostic labels: No Finding (healthy), Enlarged Cardiomediastinum, Cardiomegaly, Lung Opacity, Lung Lesion, Edema, Consolidation, Pneumonia, Atelectasis, Pneumothorax, Pleural Effusion, Pleural Other, Fracture, and Support Devices.

CheXpert Plus is a dataset of paired reports and images, comprising 223,228 unique radiology report-chest X-ray pairs. These pairs include both posteroanterior-only image-text report pairs and posteroanterior-lateral image-text report pairs. The report section of the dataset consists of up to eleven parts: Narrative, Clinical History, History, Comparison, Technique, Procedure Comments, Findings, Impression, End of Impression, Summary, and Accession Number. This dataset has the same diagnostic labels as MIMIC-CXR.

The retrospective data were sourced from 27 medical centers in China, collectively referred to as CXR-27. A total of 12,396 image-text report pairs with clinical history were included, categorized by image type into 10,260 posteroanterior-only cases, 1,632 posteroanterior-lateral cases, and 504 cases with historical chest X-ray images. They were randomly split into a fine-tuning set and a test set at a ratio of 9:1.

Supplementary Note 3 Prospective medical centers.

Union Hospital, Tongji Medical College, Huazhong University of Science and Technology; The First Affiliated Hospital of Zhengzhou University; The First Affiliated Hospital of Henan University of Science and Technology

Supplementary Note 4 Data cleaning of MIMIC-CXR and CheXpert Plus datasets.

1. MIMIC-CXR

It was divided into a fine-tuning set and a test set in accordance with official classification standards. Only posteroanterior chest radiographs were included, and the reports must contain the "FINDINGS" module or the "FINDINGS+IMPRESSION" module to form posteroanterior image-text report pairs. The DeepSeek-V3 671B model was employed to clean the reports, eliminating redundant content such as perspective information, time information, underlines, and before-and-after comparisons. Eventually, 162,105 fine-tuning image-text report pairs and 2,365 test image-text report pairs were obtained.

2. CheXpert Plus

Based on official classification, only the fine-tuning set was retained, which included 190,753 posteroanterior-only image-text report pairs and 31,350 posteroanterior-lateral image-text report pairs.

Supplementary Note 5 Automated labeling tool for chest x-ray reports.

To ensure a fair comparison with previously published research results on the MIMIC-CXR dataset, we used the CheXpert labeling tool[2] to extract binary classification labels (indicating radiographic findings) from radiology reports to evaluate our model's performance.

To more accurately extract binary classification labels for radiographic findings, we developed an automated annotation tool based on DeepSeek for extracting structured labels of 14 key clinical findings (such as pneumonia, pleural effusion, etc.) from chest X-ray reports. The tool analyzes free-text reports by calling the deepseek-chat model and outputs JSON-formatted results indicating the presence or absence (0/1) of each finding, with support for batch processing of CSV files. The tool incorporates error-handling mechanisms and employs incremental saving to ensure data processing reliability. This tool significantly improves dataset labeling efficiency and provides standardized labels for model training and validation.

A comparison of the F1 scores between the two labeling tools on the CXR-27 test set is as follows:

Label	CheXpert_F1	DS_F1
Enlarged Cardiomediastinum	0.1197	0.9524
Cardiomegaly	0.5434	1.0000
Lung Opacity	0.6414	0.9959
Lung Lesion	0.6361	0.9837
Consolidation	0.3333	0.9677
Pneumonia	0.1255	0.9920
Atelectasis	0.4330	1.0000
Pneumothorax	0.8627	1.0000
Pleural Effusion	0.8108	1.0000
Pleural Other	0.5468	0.9948
Fracture	0.5909	0.9811
Support Devices	0.9511	0.9957
No Finding	0.1391	0.9977

A comparison of the F1 scores between the two labeling tools on the MIMIC-CXR test set is as follows:

Label	CheXpert_F1	DS_F1
Enlarged Cardiomediastinum	0.9016	0.9606
Cardiomegaly	0.9507	0.9735
Lung Opacity	0.8828	0.9329
Lung Lesion	0.8438	0.9552
Consolidation	0.8136	0.9103
Pneumonia	0.8219	0.8864
Atelectasis	0.8542	0.9608
Pneumothorax	0.8057	0.9231
Pleural Effusion	0.8800	0.9167
Pleural Other	0.9405	0.9591
Fracture	0.9565	0.9231
Support Devices	0.9032	0.9697
No Finding	0.8675	0.9415

On the MIMIC-CXR test set, both annotation tools achieve relatively high accuracy; however, on the CXR-27 test set, the accuracy of the DeepSeek automated annotation tool is significantly higher than that of the CheXpert annotation tool for certain disease manifestations.

The Prompt of the automated annotation tool based on DeepSeek is as follows:

System Prompt: You are a professional radiologist specializing in chest X-ray diagnosis. Please carefully analyze the content of the report and determine whether specific radiological findings are present.

Prompt: Based on the following medical chest X-ray report, determine whether the following 14 findings are present. Mark "1" if present and "0" if absent. Only reply with a JSON dictionary containing each finding and its corresponding 0 or 1.

Medical Report: [report]

Findings to check: no finding, enlarged cardiomediastinum, cardiomegaly, lung opacity, lung lesion, edema, consolidation, pneumonia, atelectasis, pneumothorax, pleural effusion, pleural other, fracture, and support devices.

Supplementary Note 6 Multi-dimensional automated report generation metrics to evaluate report generation quality.

We employed a multi-dimensional automated metric system to evaluate report generation quality, encompassing both clinical finding recognition accuracy and text generation quality. Using CheXbert and DeepSeek annotation tools, we calculated micro-averaged/macro-averaged F1 scores for 14 disease categories (F1-14) and the top 5 most common diseases (F1-5) to assess the model's capability in identifying key radiographic findings. Rad-Graph F1 scores were adopted to quantify the accuracy of anatomical structure descriptions and their relationships.

All comparative experiments followed the original paper's parameter configurations, with open-source models tested directly and non-open-source models citing literature data.

Supplementary Note 7 Standardized Training and Assessment for Subjective Evaluation

To ensure that the 5 radiologists participating in the subjective evaluation (all with 8-15 years of professional experience) have a consistent understanding of the standards for each evaluation dimension, standardized training was conducted prior to the evaluation. The total training duration was 3 hours, specifically including 1 hour of interpretation of evaluation criteria (including scoring criteria and examples) and 2 hours of group case practice. During the practice, the radiologists performed report quality scoring and consistency scoring (on a 1-5 scale) for 10 AI-generated reports and 10 clinically published reports, and discussed scoring differences to unify the standards. All evaluators participated in the entire training process and passed the post-training assessment to ensure they possessed unified evaluation competence.

The assessment scheme is as follows: Each of the 5 evaluators rated 20 reports (10 AI generated reports and 10 clinically published reports, randomly shuffled), and the Kendall’s W coefficient was calculated. The strength of agreement between the observers was classified as poor ($W < 0.2$), fair ($0.2 \le W < 0.4$), moderate ($0.4 \le W < 0.6$), strong ($0.6 \le W < 0.8$), or super ($0.8 \le W < 1.0$).

The assessment results are as follows:

	Kendall's W	P value
Report Quality Score	0.871	<0.001
Agreement Score	0.845	<0.001

Supplementary Note 8 Radiology report quality scoring criteria (5-point likert scale).

Score	Criteria	Example
5	Flawless structure, terminology, and diagnostic reasoning; All findings precisely described with clinical context; Diagnosis matches gold standard perfectly	FINDINGS: The thoracic cage is bilaterally symmetrical. Bilateral lung fields show increased, disorganized interstitial markings; patchy increased density with blurred margins is seen in the lower lung fields. Bilateral diaphragmatic surfaces are smooth; costophrenic angles are slightly blunted. The cardiac silhouette is normal in size and shape.<br>IMPRESSION: Bilateral lower lung field exudative changes; Possible minimal bilateral pleural effusion or pleural thickening; Recommend short-term follow-up chest X-ray to assess lesion resolution.
4	Minor formatting issues or slight terminology deviations; 1-2 non-critical findings described less precisely; Diagnosis correctly but may lack nuance	FINDINGS: The thoracic cage is bilaterally symmetrical. Bilateral lung markings are increased and slightly disorganized; patchy areas of increased density are seen in the bilateral lower lung fields, with somewhat ill-defined margins. Bilateral diaphragmatic surfaces are smooth, and the costophrenic angles are slightly blunted. The cardiac silhouette is roughly normal in size and shape.<br>IMPRESSION: Bilateral lower lung field exudative changes; possible minimal bilateral pleural effusion; clinical correlation is recommended.
3	Basic sections present (findings/impression); Some findings unclear/partially wrong; The diagnostic direction is unclear or has deviations	FINDINGS: The thoracic cage is generally normal. Bilateral lung markings are generally unremarkable. Bilateral diaphragmatic surfaces are relatively smooth, and the costophrenic angles appear slightly blunted. The cardiac silhouette is generally normal in size.<br>IMPRESSION: Possible bilateral pleural effusion; Further clarification of the diagnosis is required.
2	Missing major sections or confusing flow; Multiple errors affecting interpretation; Diagnosis questionable	FINDINGS: The thoracic cage shows no obvious abnormalities. Small patchy high-density shadows are noted in both lungs (with unclear nature). Diaphragms were not carefully evaluated; the cardiac silhouette appears slightly enlarged?<br>IMPRESSION: Possible abnormalities in both lungs; Diaphragms were not carefully evaluated; The cardiac silhouette is enlarged.

1	Unreadable or medically dangerous errors; Critical findings missed/misrepresented; Diagnosis contradicts imaging	The thoracic cage is bilaterally symmetrical. Bilateral lung markings are markedly increased and disorganized. An extensive area of suspected consolidation is noted in the left lobe of the lung.

Supplementary Note 9 RADPEER scoring criteria

The RADPEER system, established by the American College of Radiology (ACR), is a peer review program designed to evaluate the interpretation accuracy of radiologists. Using the RADPEER scoring system, the degree of concordance is assessed with discrepancies and agreements graded according to the following criteria:

Category 1: Clinically significant discrepancy

Category 2: Clinically insignificant discrepancy

Category 3: Understandable clinically significant miss (excusable given case complexity)

Category 4: Understandable clinically insignificant miss (excusable due to subtle findings, case complexity, or diagnostic difficulty)

Category 5: Complete agreement

Supplementary Note 10 Sample size calculation

Based on preliminary trial data (n=30), the mean difference in imaging report scores between the AI-assisted group and the SCP group was 0.207 points (standard deviation $\sigma\_diff=0.872$). To detect this difference (two-tailed $\alpha=0.05$, power=0.90) while accounting for a 30% dropout rate, the calculated required sample size was 270 cases. To ensure robust results capable of detecting statistically significant differences across multiple evaluation metrics - including report quality scores, pairwise preference testing, consistency assessments, and reading time - the study plans to enroll 300 patients.

To determine the physician sample size while controlling for inter-physician variability in diagnostic performance assessment, we adopted a framework similar to that of a cluster randomized trial. The core formula was defined as $N=2/\Delta^2\times(Z_{1-\alpha/2}+Z_{1-\beta})^2\cdot\sigma^2\times[1+(m-1)\times ICC]$, where inter-physician variance $\sigma^2=0.66$, the minimum clinically significant difference $\Delta=0.5$ points, and the intraclass correlation coefficient ICC=0.15. Substituting parameters yielded N≈8.2 per group. We ultimately enrolled 10 physicians per group (rounded up with 20% buffer to account for attrition), ensuring 80% statistical power.

Supplementary Figure 1 Confusion matrix evaluation of large model-generated radiology reports.

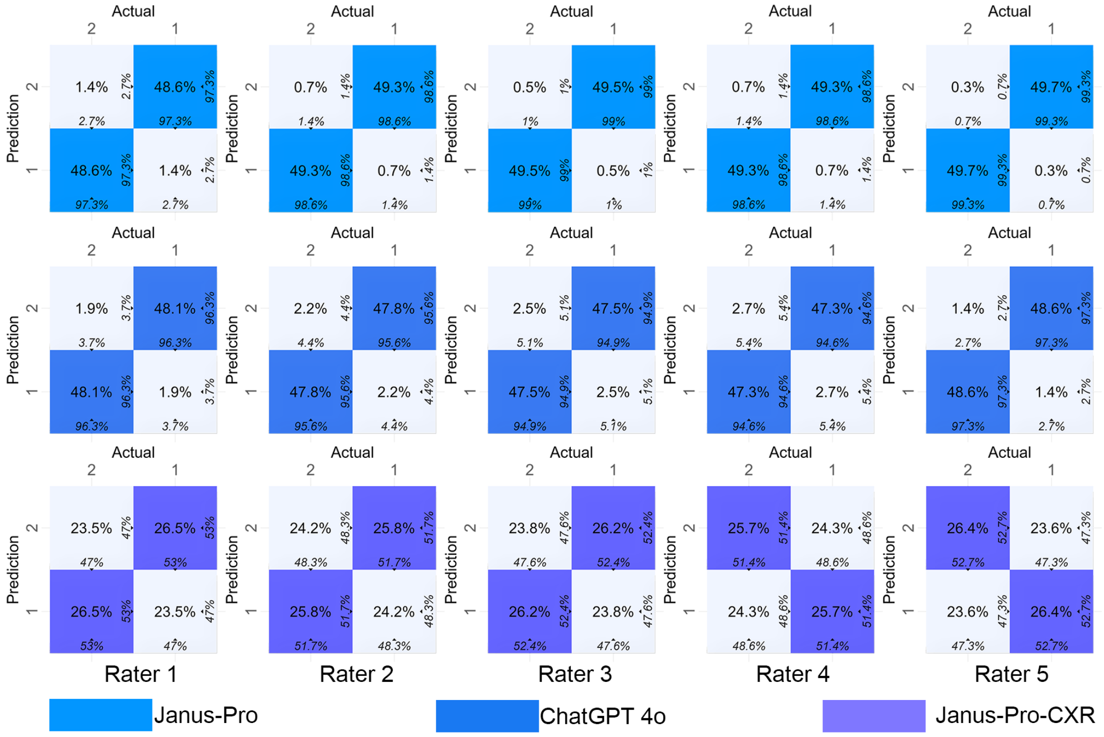

Five experts independently evaluated the style and formatting of AI-generated reports by comparing them against published reference reports, while deliberately excluding consideration of content accuracy.

Supplementary Figure 2 Receiver operating characteristic (ROC) curves (AUC<0.8) of 14 predictive features in the CXR-27 Test Set.

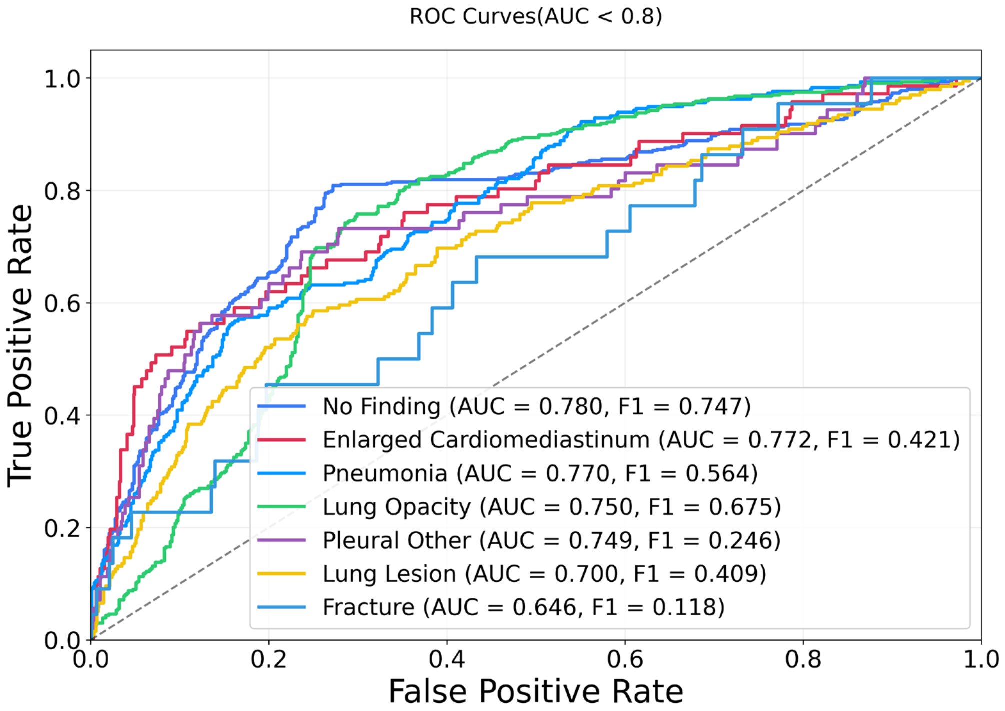

The ROC curves were constructed using probability thresholds ranging continuously from 0 to 1. Notably, the edema classification was excluded from analysis due to insufficient positive cases, as such limited sample size would yield non-representative ROC curve characteristics.

Supplementary Figure 3 Prospective Validation for Multi-Image Input Scenarios Based on 50 Patients with Posteroanterior and Lateral Chest X-rays.

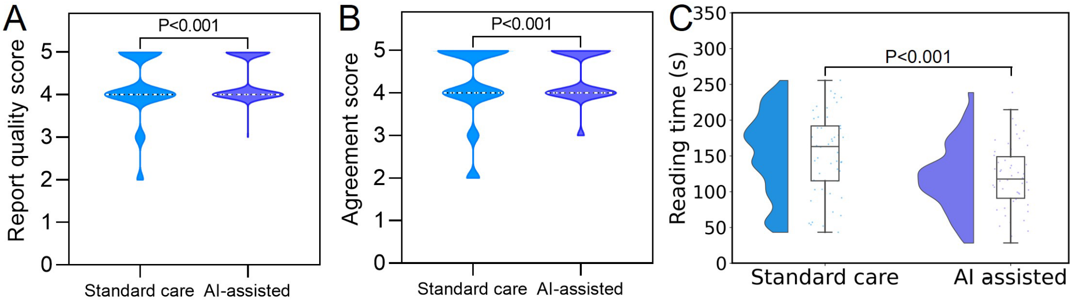

(A) Report quality scores for the standard care group and the AI assisted group in the prospective study (n=50). (B) Report agreement scores for the standard care group and the AI assisted group in the prospective study (n=50). The paired t-tests were used in (A) and (B). Each violin's width reflects data point density at respective values; white dashed lines show the median, black dashed lines the interquartile range (25th–75th percentiles). (C) Reading time for the standard care group and the AI assisted group in the prospective study (n=50). The paired t-test was used and the center line corresponds to the median, and the box is delineated by the first and third quartiles.

Supplementary Figure 4 Interactive Interface for Professional Medical Imaging Analysis.

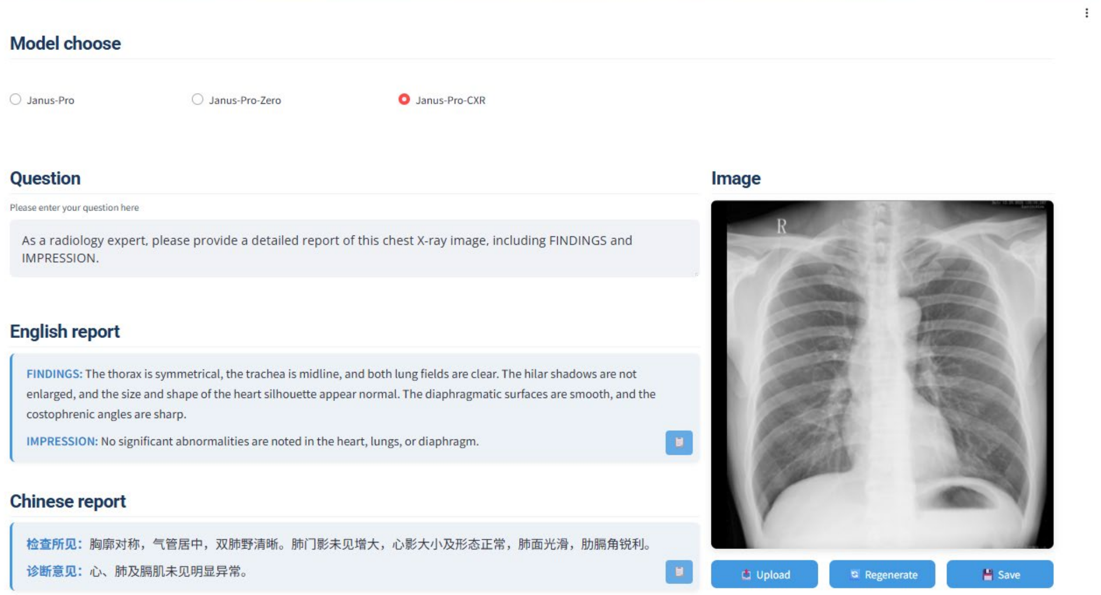

Users can switch between different models and request a detailed report on a chest X - ray image from the perspective of a radiology expert in the "Question" box, including FINDINGS and IMPRESSION. The "Answer" box provides the analysis results in English, and the "Translation" box offers the corresponding Chinese translation. The chest X - ray image is displayed on the right side of the interface.

Supplementary Table 1 Baseline characteristics of the retrospective study.

Characteristic	Fine-tuning			Testing	
	MIMIC-CXR (n=162,105)	CheXpert Plus (n=222,103)	CXR-27 (n=11,156)	MIMIC-CXR (n=2,365)	CXR-27 (n=1,240)
Age, years (mean ± s.d.)	-	-	40.2±25.7	-	38.9±29.2
Age class, n (%)					
<18	-	-	1907 (17.1%)	-	176 (14.2%)
18-60	-	-	6716 (60.2%)	-	671 (54.1%)
>60	-	-	2533 (22.7%)	-	393 (31.7%)
Sex, n (%)					
male	-	-	6236 (55.9%)	-	679 (54.8%)
female	-	-	4920 (44.1%)	-	561 (45.2%)
Disease, n (%)					
No Finding	69164 (42.7%)	22277 (10.0%)	4611 (41.3%)	164 (6.9%)	484 (39.0%)
Support Devices	29586 (18.3%)	115294 (51.9%)	1331 (11.9%)	1328 (56.2%)	165 (13.3%)
Pleural Effusion	27002 (16.7%)	85643 (38.6%)	1266 (11.3%)	898 (38.0%)	141 (11.4%)
Lung Opacity	32730 (20.2%)	104921 (47.2%)	5053 (45.3%)	1138 (48.1%)	570 (46.0%)
Pneumonia	10691 (6.6%)	5997 (2.7%)	2545 (22.8%)	326 (13.8%)	289 (23.3%)
Cardiomegaly	20423 (12.6%)	26830 (12.1%)	997 (8.9%)	844 (35.7%)	109 (8.8%)
Lung Lesion	4045 (2.5%)	9128 (4.1%)	1763 (15.8%)	237 (10.0%)	194 (15.6%)
Enlarged Cardiomediastinum	3023 (1.9%)	10728 (4.8%)	440 (3.9%)	545 (23.0%)	55 (4.4%)
Pneumothorax	5385 (3.3%)	19345 (8.7%)	631 (5.7%)	74 (3.1%)	77 (6.2%)
Atelectasis	24811 (15.3%)	33182 (14.9%)	660 (5.9%)	973 (41.1%)	78 (6.3%)
Pleural Other	1037 (0.6%)	3509 (1.6%)	695 (6.2%)	160 (6.8%)	73 (5.9%)
Consolidation	4032 (2.5%)	14703 (6.6%)	82 (0.7%)	306 (12.9%)	15 (1.2%)
Fracture	2874 (1.8%)	8995 (4.0%)	325 (2.9%)	157 (6.6%)	31 (2.5%)
Edema	14635 (9.0%)	51928 (23.4%)	10 (0.1%)	688 (29.1%)	0

The retrospective data from 27 medical centers in China were named CXR-27, which was divided into a fine-tuning set and a test set at a ratio of 9:1.

Supplementary Table 2 Baseline characteristics of prospective study participants.

Characteristic	Value (n = 296)
Age, years (mean ± s.d.)	39.1±26.3
Age class, n (%)	
<18	86 (29.1%)
18-60	125 (42.2%)
>60	85 (28.7%)
Sex, n (%)	
male	157 (53.0%)
female	139 (47.0%)
Disease, n (%)	
No Finding	122 (41.2%)
Support Devices	24 (8.1%)
Pleural Effusion	21 (7.1%)
Lung Opacity	132 (44.6%)
Pneumonia	93 (31.4%)
Cardiomegaly	28 (9.5%)
Lung Lesion	66 (22.3%)
Enlarged Cardiomediastinum	33 (11.1%)
Pneumothorax	8 (2.7%)
Atelectasis	9 (3.0%)
Pleural Other	22 (7.4%)
Consolidation	7 (2.4%)
Fracture	4 (1.4%)
Edema	0

Supplementary Table 3 Inter-rater reliability verification in the prospective study.

		Standard care	AI-assisted
Report Quality Score	Kendall's W	0.738	0.752
	P value	<0.001	<0.001
Agreement Score	Kendall's W	0.699	0.586
	P value	<0.001	<0.001

Kendall's W coefficient was used to assess inter-rater agreement. The null hypothesis (W = 0, random rankings) was rejected if $P < 0.05$ (with two-sided Friedman's χ^2 test). The strength of agreement between observers was classified as poor ($W < 0.2$), fair ($0.2 \leq W < 0.4$), moderate ($0.4 \leq W < 0.6$), strong ($0.6 \leq W < 0.8$), or excellent ($0.8 \leq W < 1.0$).

Supplementary Table 4 An example of AI improving junior radiologists' reporting accuracy.

Image	Report by senior radiologists reviewed and released	Report by a junior radiologist (Standard care group)	AI-generated report	AI-assisted report
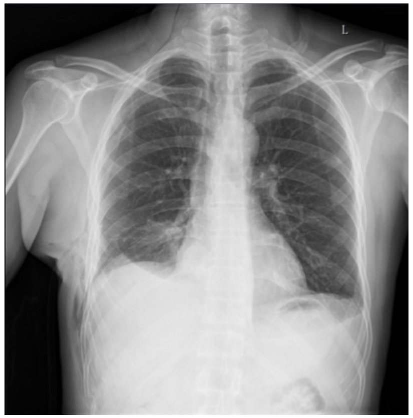	**Findings:** Postoperative changes in the right lung with elevated right hemidiaphragm. A drainage tube is seen in the right thoracic cavity, along with minimal subcutaneous emphysema in the right chest wall. Patchy slightly high-density shadows and linear opacities are observed in the right lower lung field. The cardiac silhouette is not enlarged, and bilateral costophrenic angles are blunted. **Impression:** 1. Postoperative status of the right lung with elevated right hemidiaphragm, presence of a drainage tube in the right thoracic cavity, and minimal subcutaneous emphysema in the right chest wall. 2. Patchy slightly high-density shadows and linear opacities in the right lower lung field, likely postoperative changes; follow-up imaging is recommended. 3. Bilateral minimal pleural effusion. 4. A few high-density nodular shadows are noted in both lung fields; a CT examination is recommended.	**Findings:** Postoperative changes are noted in the right lung, with a drainage tube visible in the right pleural cavity and pneumothorax observed along the right chest wall. The trachea and mediastinum are centrally located, with no significant abnormalities in the size or shape of the cardiac silhouette. Both diaphragmatic surfaces are smooth, and the costophrenic angles are sharp bilaterally. **Impression:** Postoperative changes in the right lung, presence of a drainage tube in the right pleural cavity, and pneumothorax along the right chest wall. A few high-density nodular shadows are noted in both lung fields; a CT examination is recommended.	**Findings:** Postoperative changes in the right lung with a drainage tube visible in the right thoracic cavity, pneumatosis in the right chest wall, and a few hazy linear and patchy opacities in the lower right lung field. The trachea and mediastinum are midline. The cardiac silhouette shows no significant abnormalities in size or shape. Both diaphragmatic surfaces are smooth, with blunting of the left costophrenic angle and a small amount of gas in the right thoracic cavity. **Impression:** 1. Postoperative changes in the right lung with a drainage tube visible in the right thoracic cavity, pneumatosis in the right chest wall, and a few hazy linear and patchy opacities in the lower right lung field, suggestive of inflammatory changes/subsegmental atelectasis. 2. Small amount of left pleural effusion. 3. Small amount of right pleural effusion.	**Findings:** Postoperative changes in the right lung with a drainage tube visible in the right thoracic cavity, pneumothorax along the right chest wall, and a few hazy linear and patchy opacities in the right lower lung field. The trachea and mediastinum are midline. The cardiac silhouette shows no significant abnormalities in size or shape. Both diaphragmatic surfaces are smooth, with blunting of the left costophrenic angle and a small amount of gas in the right thoracic cavity. **Impression:** 1. Postoperative changes in the right lung with a drainage tube visible in the right thoracic cavity, pneumothorax along the right chest wall, and a few hazy linear and patchy opacities in the right lower lung field, possibly due to inflammatory changes. 2. Bilateral small pleural effusions.

Supplementary Table 5 Inter-rater reliability verification in the retrospective study

		Janus-Pro	ChatGPT 4o	Janus-Pro CXR
Report Quality Score	Kendall's W	0.622	0.716	0.725
	P value	<0.001	<0.001	<0.001
Agreement Score	Kendall's W	0.591	0.680	0.577
	P value	<0.001	<0.001	<0.001

Kendall's W coefficient was used to assess inter-rater agreement. The null hypothesis (W = 0, random rankings) was rejected if $P < 0.05$ (with two-sided Friedman's χ^2 test). The strength of agreement between observers was classified as poor ($W < 0.2$), fair ($0.2 \leq W < 0.4$), moderate ($0.4 \leq W < 0.6$), strong ($0.6 \leq W < 0.8$), or excellent ($0.8 \leq W < 1.0$).

Supplementary Table 6 Automated report generation metrics.

(A) Automated report generation metrics on MIMIC-CXR Test Set.

Model	Year	Size	CheXbert label				RadGraph
			"uncertain" as negative				
			Micro-avg		Macro-avg		
			F1-14	F1-5	F1-14	F1-5	F1
**OURS**	2025	1.5B	<u>59.9</u>	**63.4**	<u>42.3</u>	**55.1**	25.8
CheXagent[3]	2024	3B	58	62.5	**44.9**	<u>55</u>	26.6
MAIRA-2[4]	2024	7B	58.1	59.1	41.6	50.4	**34.6**
M4CXR[5]	2024	\	**60.6**	<u>61.8</u>	40	49.5	21.8
LLaVA-Rad[6]	2024	7B	57.3	57.4	39.5	47.7	<u>29.4</u>
GPT-4V[7]	\	\	35.5	25.8	20.4	19.6	13.2
HealthGPT[8]	2025	3.8B	21.2	22.2	13.3	16.6	14.1
FlamingoCXR[9]	2024	3B	\	\	\	\	20.5
PromptMRG[10]	2024	<1B	15.3	6	7.84	3.53	20
Med-PaLM M[11]	2023	84B	53.6	57.9	39.8	51.6	26.7
LLaVA-Med[12]	2023	7B	27.2	22	15.5	16.6	6.5
R2GenGPT[13]	2023	7B	\	\	\	\	\

(B) Automated report generation metrics on CXR-27 Test Set.

Model	Year	Size	DeepSeek label				RadGraph
			"uncertain" as positive				
			Micro-avg		Macro-avg		
			F1-14	F1-5	F1-14	F1-5	F1
**OURS**	2025	1.5B	**60.5**	**59**	**49.1**	**57**	**58.6**
**OURS-Zero**	2025	1.5B	<u>47.6</u>	<u>30.6</u>	<u>35</u>	<u>32.9</u>	15.9
CheXagent[3]	2024	3B	42.8	26.4	33.3	28.6	15.4
HealthGPT[8]	2025	3.8B	35.2	21.9	15.3	14.3	<u>20.9</u>
CvT-21DistillGPT2[14]	2023	<1B	38.4	16.9	20.6	17.2	11

(A) Automated report generation metrics on the MIMIC test set. CheXbert labeling tool was used for annotation (uncertain labels were treated as negative). Top 5 conditions in MIMIC test set: Atelectasis, Cardiomegaly, Edema, Consolidation, Pleural Effusion. (B) Automated report generation metrics on the CXR-27 test set. DeepSeek labeling tool was employed, testing the open-source models (parameter configurations strictly followed original papers). Top 5 conditions in CXR-27 test set: Support Devices, Pleural Effusion, Lung Opacity, Pneumonia, Lung Lesion. The top performer is shown in bold, while the runner-up is underlined. F1-14 represents F1 scores across 14 diseases; F1-5 covers the five most common conditions. B denotes model parameters in billions (1B = 1 billion parameters), where typically fewer parameters indicate lower model development and operational costs.

Supplementary Table 7 Model performance evaluation using F1 scores on the CXR-27 test set.

Model	F1													
	ECm.	Cmgl.	Opac.	Les.	Edema	Cnsl.	Pna.	Atel.	Pmtx.	Eff.	P.O.	Frac.	Dev.	NoF.
**OURS**	**42.1**	**47.4**	**67.5**	**40.9**	0.0	27.8	**56.4**	**52.6**	52.6	**66.7**	24.6	**11.8**	**72.7**	**74.7**
**OURS-Zero**	24.7	36.8	35.2	13.5	0.0	**28.6**	18.1	42.9	57.4	58.7	0.1	1.0	60.8	70.7
CheXagent[3]	7.9	36.2	29.7	18.9	0.0	25.0	16.4	35.9	**60.0**	55.5	**31.4**	6.5	39.7	69.6
HealthGPT[8]	10.9	13.5	50.0	29.8	0.0	4.3	44.1	8.8	7.7	10.5	13.4	3.8	16.8	19.8
CvT-21DistillGPT2[14]	11.2	11.3	53.0	31.8	0.0	4.1	43.5	4.3	7.3	11.5	13.1	4.2	14.5	5.3

The open-source models were tested with parameter configurations strictly adhering to the original papers, where the top-performing model is presented in **bold** and the second-best model is indicated with underscores. ECm., Enlarged Cardiomediastinum; Cmgl., Cardiomegaly; Opac., Lung Opacity; Les., Lung Lesion; Cnsl., Consolidation; Pna., Pneumonia; Atel., Atelectasis; Pmtx., Pneumothorax; Eff., Pleural Effusion; P.O., Pleural Other; Frac., Fracture; Dev., Support Devices; NoF., No Finding.

Supplementary Table 8 An example of historical chest radiographs and current chest radiographs as model inputs.

<table>
<tr><th>Chest radiographs</th><th>Reference report</th><th>AI-generated report</th></tr>
<tr><td>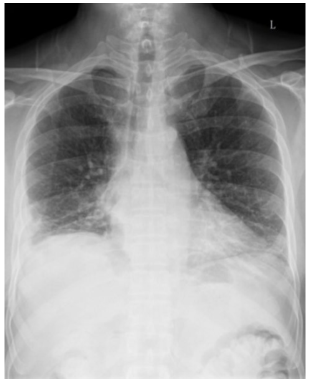
Current chest radiograph</td><td rowspan="2">Findings: 1. Postoperative changes in the right lung with minimal residual pneumothorax/pleural air on the right side, showing improvement compared to previous findings. 2. Linear and patchy dense opacities in the lower lung fields bilaterally, more pronounced on the right, suggestive of inflammation/atelectasis, showing improvement compared to previous findings. 3. Minimal right pleural effusion; the left costophrenic angle appears largely clear. 4. The cardiac silhouette is slightly prominent.
Impression: Findings as described above.</td><td rowspan="2">Findings: 1. Postoperative changes in the right lung, with minimal pneumothorax on the right side and minimal subcutaneous emphysema in the right chest wall, with improvement compared to the previous examination. 2. Linear and patchy opacities in the lower lung fields bilaterally, suggestive of inflammatory changes/segmental atelectasis, showing improvement compared with the previous examination. 3. Slightly thickened pleura in the right lower lung field, with minimal pleural effusion. Follow-up or CT examination is recommended. 4. Slightly thickened and blurred lung markings in both lungs, with a few linear opacities in the lower lung fields bilaterally, possibly due to chronic inflammatory changes. 5. Slightly enlarged cardiac silhouette.
Impression: Findings as described above.</td></tr>
<tr><td>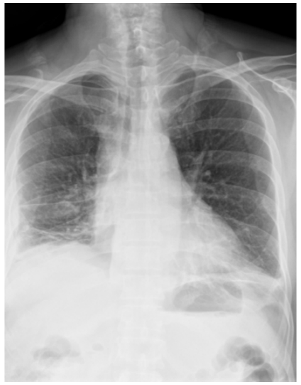
Historical chest radiograph</td></tr>
</table>

Supplementary Table 9 An example of posteroanterior and lateral chest radiographs as model inputs.

Chest radiographs	Reference report	AI-generated report
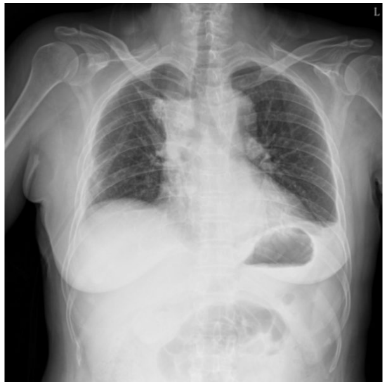<br>Posteroanterior view<br>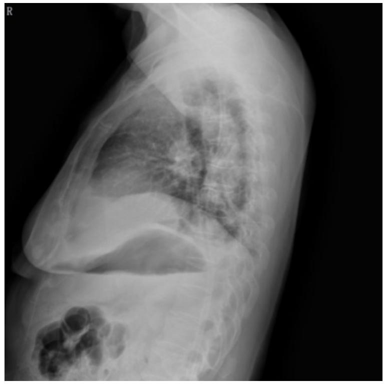<br>Lateral view	**Findings:** 1. Postoperative changes in the right lung; presence of a right thoracic drainage tube; enlargement of the right hilum; patchy high-density opacity in the upper and middle zones of the right lung, follow-up recommended. 2. Minimal pleural effusion bilaterally. 3. Normal cardiac silhouette.<br>**Impression:** Findings as described above. Clinical correlation and follow-up are advised.	**Findings:** 1. Postoperative changes in the right lung, with enlargement of the right hilum and patchy opacities in the right lung field. A drainage tube is visible in the right pleural cavity. 2. Slightly thickened lung markings in both lungs, with a few linear and patchy opacities in the left lower lung field, suggesting possible mild inflammatory changes or subsegmental atelectasis. 3. Enlarged cardiac silhouette. 4. Possible minimal bilateral pleural effusion.<br>**Impression:** postoperative changes in the right lung. Clinical correlation and follow-up/CT examination are recommended.

Supplementary Table 10 An example of the model's suboptimal performance.

Chest radiograph	Reference report	AI-generated report
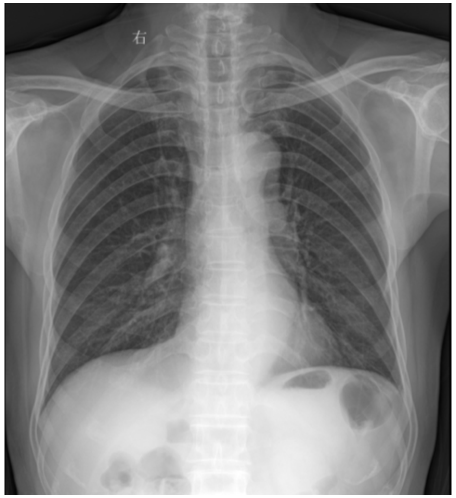	**Findings:** the thorax is symmetrical, the trachea is midline, and both lung fields are clear. The hilar shadows are not enlarged. The aorta is tortuous. The cardiac silhouette is normal in size and configuration. Both diaphragmatic surfaces are smooth, and the costophrenic angles are sharp.<br>**Impression:** tortuosity of the aorta.	**Findings:** 1. The trachea is slightly deviated. Increased lung markings with partially blurred margins are observed in both lungs, along with scattered small punctate, nodular, and patchy opacities. 2. The heart shows no significant enlargement. The aorta is tortuous, calcified, and locally dilated. 3. The diaphragmatic surfaces are smooth bilaterally, and the costophrenic angles are sharp.<br>**Impression:** - slight deviation of the trachea. - possible bronchitis and minor inflammatory lesions in both lungs, with small pulmonary nodules. - tortuosity, calcification, and localized dilation of the aorta. - clinical correlation and further CT examination if necessary are recommended.

References

1. Johnson, A.E.W., *et al.* MIMIC-CXR, a de-identified publicly available database of chest radiographs with free-text reports. *Scientific Data* **6**, 317 (2019).
2. Smit, A., *et al.* Combining Automatic Labelers and Expert Annotations for Accurate Radiology Report Labeling Using BERT. 1500-1519 (Association for Computational Linguistics, Online, 2020).
3. Chen, Z., *et al.* A Vision-Language Foundation Model to Enhance Efficiency of Chest X-ray Interpretation. arXiv:2401.12208 (2024).
4. Bannur, S., *et al.* MAIRA-2: Grounded Radiology Report Generation. arXiv:2406.04449 (2024).
5. Park, J., Kim, S., Yoon, B., Hyun, J. & Choi, K.J.a.e.-p. M4CXR: Exploring Multi-task Potentials of Multi-modal Large Language Models for Chest X-ray Interpretation. arXiv:2408.16213 (2024).
6. Zambrano Chaves, J.M., *et al.* Towards a clinically accessible radiology foundation model: open-access and lightweight, with automated evaluation. arXiv:2403.08002 (2024).
7. Yang, Z., *et al.* The Dawn of LMMs: Preliminary Explorations with GPT-4V(ision). arXiv:2309.17421 (2023).
8. Lin, T., *et al.* HealthGPT: A Medical Large Vision-Language Model for Unifying Comprehension and Generation via Heterogeneous Knowledge Adaptation. arXiv:2502.09838 (2025).
9. Tanno, R., *et al.* Collaboration between clinicians and vision–language models in radiology report generation. *Nat Med* **31**, 599-608 (2025).
10. Jin, H., Che, H., Lin, Y. & Chen, H.J.a.e.-p. PromptMRG: Diagnosis-Driven Prompts for Medical Report Generation. arXiv:2308.12604 (2023).
11. Tu, T., *et al.* Towards Generalist Biomedical AI. arXiv:2307.14334 (2023).
12. Dong, X., *et al.* LLaDA-MedV: Exploring Large Language Diffusion Models for Biomedical Image Understanding. arXiv:2508.01617 (2025).
13. Wang, Z., Liu, L., Wang, L. & Zhou, L.J.a.e.-p. R2GenGPT: Radiology Report Generation with Frozen LLMs. arXiv:2309.09812 (2023).
14. Nicolson, A., Dowling, J. & Koopman, B. Improving chest X-ray report generation by leveraging warm starting. *Artificial Intelligence in Medicine* **144**, 102633 (2023).
