# Supplementary material for: A DeepSeek-powered AI system for automated chest radiograph interpretation in clinical practice
Source: Nat Commun. 2026 May 7;17:6141. doi: 10.1038/s41467-026-72680-6 (PMC13365505; doi:10.1038/s41467-026-72680-6)
Supplement: Supplementary file 2 — Reporting summary [file 41467_2026_72680_MOESM2_ESM.pdf]

nature portfolio

Corresponding author(s): Dacheng Tao, Yongchao Xu, Chuansheng Zheng, Huangxuan Zhao, Bo Du

Last updated by author(s): Mar 1, 2026

Reporting Summary

Nature Portfolio wishes to improve the reproducibility of the work that we publish. This form provides structure for consistency and transparency in reporting. For further information on Nature Portfolio policies, see our Editorial Policies and the Editorial Policy Checklist.

Statistics

For all statistical analyses, confirm that the following items are present in the figure legend, table legend, main text, or Methods section.

n/a	Confirmed	
☐	☒	The exact sample size (n) for each experimental group/condition, given as a discrete number and unit of measurement
☐	☒	A statement on whether measurements were taken from distinct samples or whether the same sample was measured repeatedly
☐	☒	The statistical test(s) used AND whether they are one- or two-sided<br>*Only common tests should be described solely by name; describe more complex techniques in the Methods section.*
☐	☒	A description of all covariates tested
☐	☒	A description of any assumptions or corrections, such as tests of normality and adjustment for multiple comparisons
☐	☒	A full description of the statistical parameters including central tendency (e.g. means) or other basic estimates (e.g. regression coefficient) AND variation (e.g. standard deviation) or associated estimates of uncertainty (e.g. confidence intervals)
☐	☒	For null hypothesis testing, the test statistic (e.g. F, t, r) with confidence intervals, effect sizes, degrees of freedom and P value noted<br>*Give P values as exact values whenever suitable.*
☒	☐	For Bayesian analysis, information on the choice of priors and Markov chain Monte Carlo settings
☒	☐	For hierarchical and complex designs, identification of the appropriate level for tests and full reporting of outcomes
☐	☒	Estimates of effect sizes (e.g. Cohen's d, Pearson's r), indicating how they were calculated

Our web collection on statistics for biologists contains articles on many of the points above.

Software and code

Policy information about availability of computer code

Data collection	No software was used to collect the data.
Data analysis	Statistical analysis was performed using R software (version 4.2.1). The code for the model and the DeepSeek labeling tool can be available from the URL (https://github.com/ZrH42/Janus-Pro-CXR)

For manuscripts utilizing custom algorithms or software that are central to the research but not yet described in published literature, software must be made available to editors and reviewers. We strongly encourage code deposition in a community repository (e.g. GitHub). See the Nature Portfolio guidelines for submitting code & software for further information.

Data

Policy information about availability of data

All manuscripts must include a data availability statement. This statement should provide the following information, where applicable:

- Accession codes, unique identifiers, or web links for publicly available datasets
- A description of any restrictions on data availability
- For clinical datasets or third party data, please ensure that the statement adheres to our policy

All the data supporting the findings of this study are available within this article and its Supplementary Information. Any additional information can be obtained from corresponding authors upon request. Source data are provided with this paper.

Research involving human participants, their data, or biological material

Policy information about studies with human participants or human data. See also policy information about sex, gender (identity/presentation), and sexual orientation and race, ethnicity and racism.

Reporting on sex and gender	This study did not perform separate analyses by sex.
Reporting on race, ethnicity, or other socially relevant groupings	Not applicable.
Population characteristics	The baseline characteristics of patients in the retrospective study are presented in Supplementary Table 1. For the prospective study, data were sourced from three hospitals in China, with a total of 296 patients enrolled in the AI-radiologist collaboration. The baseline characteristics for the prospective study are provided in Supplementary Table 2.
Recruitment	Patients were recruited in strict accordance with inclusion and exclusion criteria under the supervision of the ethics committee.
Ethics oversight	Approval for this study was granted by the Institutional Review Boards of all participating centers, and it was duly registered with ClinicalTrials.gov (Identifier: NCT07117266).

Note that full information on the approval of the study protocol must also be provided in the manuscript.

Field-specific reporting

Please select the one below that is the best fit for your research. If you are not sure, read the appropriate sections before making your selection.

☒ Life sciences ☐ Behavioural & social sciences ☐ Ecological, evolutionary & environmental sciences

For a reference copy of the document with all sections, see nature.com/documents/nr-reporting-summary-flat.pdf

Life sciences study design

All studies must disclose on these points even when the disclosure is negative.

Sample size	Sample size determination for the prospective study was based on the pre-experiment results regarding report quality scores.
Data exclusions	For prospective clinical validation, the inclusion criteria included: 1) clinically suspected thoracic diseases (such as pneumonia, tuberculosis, or lung cancer) requiring CXR-assisted diagnosis, 2) patients providing written informed consent for research data use, 3) complete clinical records (including chief complaints, medical history, and laboratory test results), 4) patients with no historical chest X-ray images and no need for comparison with previous chest X-ray images, and 5) patients who underwent only posteroanterior chest X-rays without lateral chest X-rays; the exclusion criteria were: 1) substandard CXR image quality (including severe motion artifacts, over-/underexposure, or missing anatomical structures), and 2) pregnant or lactating women.
Replication	The conclusions of this study have been repeatedly confirmed in both retrospective and prospective studies.
Randomization	In the prospective study, 20 radiologists were randomly assigned to either the AI-assisted group or the standard care group in a 1:1 ratio. Randomization was conducted using a computer-generated sequence with sealed envelopes.
Blinding	The evaluating experts were unaware of the sources of the radiology reports requiring evaluation.

Reporting for specific materials, systems and methods

We require information from authors about some types of materials, experimental systems and methods used in many studies. Here, indicate whether each material, system or method listed is relevant to your study. If you are not sure if a list item applies to your research, read the appropriate section before selecting a response.

Materials & experimental systems

n/a	Involved in the study
☒	☐ Antibodies
☒	☐ Eukaryotic cell lines
☒	☐ Palaeontology and archaeology
☒	☐ Animals and other organisms
☐	☒ Clinical data
☒	☐ Dual use research of concern
☒	☐ Plants

Methods

n/a	Involved in the study
☒	☐ ChIP-seq
☒	☐ Flow cytometry
☒	☐ MRI-based neuroimaging

Clinical data

Policy information about clinical studies

All manuscripts should comply with the ICMJE guidelines for publication of clinical research and a completed CONSORT checklist must be included with all submissions.

Clinical trial registration: NCT07117266

Study protocol: The research protocol has been uploaded as supplementary material.

Data collection: Detailed information can be found in the research protocol.

Outcomes: The primary outcomes of this study included report quality scores, pairwise preference tests, agreement evaluations, and reading time for the radiology reports produced by the two groups of junior radiologists in the prospective study. Secondary outcomes comprised the report quality scores, pairwise preference tests, and agreement evaluations comparing the original reports with those generated by the model in the retrospective study.

Plants

Seed stocks: Not applicable.

Novel plant genotypes: Not applicable.

Authentication: Not applicable.
