# Supplementary material for: A DeepSeek-powered AI system for automated chest radiograph interpretation in clinical practice
Source: Nat Commun. 2026 May 7;17:6141. doi: 10.1038/s41467-026-72680-6 (PMC13365505; doi:10.1038/s41467-026-72680-6)
Supplement: Supplementary file 3 — Transparent Peer Review file [file 41467_2026_72680_MOESM3_ESM.pdf]

nature portfolio

Peer Review File

A DeepSeek-Powered AI System for Automated Chest Radiograph Interpretation in Clinical Practice

Corresponding Author: Professor Huangxuan Zhao

This manuscript has been previously reviewed at another journal. This document only contains information relating to versions considered at Nature Communications.

This file contains all reviewer reports in order by version, followed by all author rebuttals in order by version.

Version 0:

Reviewer comments:

Reviewer #1

(Remarks to the Author)
I thank the authors for their revised manuscript presenting Janus-Pro-CXR, a lightweight multimodal large language model for chest X-ray (CXR) reporting.
The authors have addressed most of my previous feedback and suggestions -- especially pursuing full fine-tuning and adding support for a second image as input -- with considerable revisions and improved their manuscript. Please find a more detailed updated review and my lingering minor concerns below.

Summary / Key Results (Updated)
The study adapts and tests a lightweight (1B parameters) multimodal large language model based on Janus-Pro, (now) fully fine-tuned in a multi-step process on MIMIC-CXR and CheXpert Plus (after revision) and a novel multicenter dataset (CXR-27), for its capability to create CXR reports. The model is evaluated in a retrospective and in a prospective setting.

A major strength of the study remains the prospective part, in which primary outcomes and results comparing AI-assisted reports vs. unassisted reports (after revision) were
- Improved report quality scores of AI-assisted reports (4.36 ± 0.50 vs. 4.12 ± 0.80 (higher is better, P < 0.001),
- Higher pairwise preference of AI-assisted reports (preference by ≥3 out of 5 readers in 54.3%),
- Higher agreement with AI-assisted reports (RADPEER score 4.30 ± 0.57 vs. 4.14 ± 0.84 (higher is better, P < 0.001)), and
- Shorter reading time (reduction of 27s (-18.3%), P < 0.001), 32.5s/-16.4% for complexer cases with >= 3 findings).

In the retrospective study, automated report generation metrics and report quality scores were assessed and the model compared favorably to comparably sized and slightly bigger multimodal report generation models and general-domain models.

Revision
In the current revision, the authors have enhanced their earlier manuscript by
- Adapting the model to accommodate two radiograph inputs (for including a prior study or a second view). This is much closer to the needs of clinical radiology and the authors find the additional incorporation of lateral views to yield significantly better performance.
- Perform full fine-tuning instead of only parameter-efficient fine-tuning with LoRA, yielding higher performance (see updated key results above)
- Adding comparisons with other recent CXR reporting models
- Adding Chexpert Plus to their training dataset
- Adding fine-tuning details, details on dataset cleaning, adding details how confidence intervals were constructed

Originality and significance (Updated)
CXR is the most widely addressed imaging modality for AI-based report generation. Many studies have used automatic metrics for radiology report generation, and a few studies have found a beneficial effect of AI-generated CXR reports on reading time (e.g., Chen et al. CheXagent https://arxiv.org/abs/2401.12208 , Hong et al.,

https://doi.org/10.1148/radiol.241646 , Hong et al. https://doi.org/10.1016/j.jacr.2025.09.015).

The present manuscript is still one of the first to present a randomized, single-blinded prospective approach. Huang et al. (https://jamanetwork.com/journals/jamanetworkopen/fullarticle/2834943) recently published a study that prospectively evaluated a CXR reporting model and found a 15.5% reduction in reporting time, in line with the observed effects presented here; however, Huang et al. did not publish their model.

Reporting time reduction is a key metric for potential adoption of radiology AI systems with real-world impact, and as such, the study will be of interest to many in radiology as well as to other specialties that use lightweight, domain-adapted, vision-grounded language models for report/note generation.

Data & methodology (Updated)
Data and methodology of this study are appropriate and mostly allow for reproduction.
- The authors use public datasets (MIMIC-CXR and CheXpert Plus), a novel multi-center dataset (CXR-27) and a prospectively collected dataset from three hospitals. Dataset creation incl. inclusion and exclusion criteria and dataset incl. patient characteristics are presented in detail in the flowchart and the supplementary materials. Previous errors in the flowcharts have been addressed.
- The model builds on a publicly available model (DeepSeek Janus-Pro).
- The associated GitHub repository holds the code and instructions for inference.
- The associated Huggingface repository holds the model weights for Janus-Pro-CXR-Zero and Janus-Pro-CXR-Final.
- (Updated) It has now been documented which DeepSeek model was used for label extraction & the prompts have been documented.

Use of statistics and treatment of uncertainties:
- Sample size calculation has been conducted using preliminary trial data.
- Statistical tests are appropriate.
- (Updated) Confidence intervals are reported, the revision also provided insight into their construction.
- (Updated) Error bars are present and have been explained more thoroughly through the revision.

Conclusions:
- The presented conclusions are supported by the results.

Suggested Improvements (Updated)

- (Updated) The presented model has now been fully fine-tuned and extended to enable more than one image input.
- (Updated) All previous minor suggestions have been addressed.
- (Updated) Training code is not provided, but the authors note that it will be made public after the article is accepted.
- (New) It’s not clear what you mean by “with testing strictly conducted in accordance with the official division standards of this dataset” (Pg. 13, l. 333f)
- (New) The new Supplementary “Table” 6 shows another suboptimal performance (model calls the effusion reduced in comparison to prior, while in reality, it has slightly progressed), consider using another example or mention this is a failure example (similar to Supplementary “Table” 8).

(Remarks on code availability)
I have not tried to run the code, but I read it; as it largely builds on DeepSeeks repositories it is expected to be executable. The associated GitHub repository holds the code and instructions for inference (specifically, generating a report based on a provided CXR image). Training code is not provided, but will be made available after publication as per the author's comments.

Model weights can be downloaded from Huggingface.

Reviewer #2

(Remarks to the Author)
This is a revised version of a manuscript that details how authors developed a DeepSeek-based system for automatic Chest X-ray interpretation. In response to multiple reviewer comments, they now describe an architecture of the model that includes multimodal inputs including lateral/historical images as well the patient’s clinical history. They have also addressed issues of unfair comparisons by including direct comparisons with MAIRA-2 and CheXagent and an experimental set-up Janus-Pro-CXR-Zero that was not fine-tuned on their internal CXR-27 test set. The revised manuscript also expanded design choices to the Discussion section and the benefits of such an open-source model for medical image interpretation. Overall, the revised manuscript addresses a majority of the core requests and concerns.

Several notable strengths at this point include:
1. A prospective trial of an open-source chest-X-ray system to aid in augmenting clinical workflows.
2. The prospective trial’s design of measuring resident+AI collaboration demonstrates a more plausible synergy and workflow optimization rather than directly pitting AI and attending radiologists as done in previous work.
3. Lightweight model architecture caters to multimodal input and clinical history, which outperforms and/or is competitive with other state-of-the-art models, offering flexibility for hospitals with limited access to GPU resources or private on-premise usage. These are also open-sourced.

Several remaining issues that I request the authors to revise or address or acknowledge as limitation as appropriate include:
1. The new architecture that integrates multimodal inputs was not tested on the prospective clinical trial which limits the generalizability of claims regarding this new architecture. It would be necessary to prospectively test this model or reword and restructure the manuscript to reflect this methodical design.
2. The authors did not include a research setting that immediately sent the AI-generated reports for attending review to benchmark against the AI-generated reports vetted by residents. Without this research setting, the authors cannot claim true diagnostic reliability and workflow efficiency.

(Remarks on code availability)
Based on my cursory review of the code (without having compiled it myself), the Readme content is adequate and the code appears to be available for download and execution. Assuming the code works as stated in the readme file, the authors have provided sufficient information to reproduce the model.

Reviewer #3

(Remarks to the Author)
I provided a mostly positive prior review of an earlier version of this manuscript, thus I focus my discussion below just on the responses to my prior review comments:

Appears study has effectively expanded physician reviewer sample size from 6 to 20 physicians to better account for clustered statistical power.

They redid a set of experiments on the CXR-27 dataset as there was some unusually poor performance of the CheXPert baseline on labeling. No clear reason why, but speculation of regional differences in clincial standards and linguistic conventions.

Added specific comparison against CXR specific models (e.g., MAIRA-2, CheXagent, ...)
Then argues towards more useful comparison to generalist models that are more available.
Then argues that clinical specialist systems is an important paradigm shift.

Can't have it both ways. Should add to discussion or limitations on how this specialist model may be good compared to generalist models, but that other specialist models could be as good but less available.

Added a Supplementary Appendex 7 to better explain how the expert panel reviewed diagnostic reports along multiple dimensions (professionalism, logical coherence, diagnostic suggestions, and other report aspects).

This response is inadequate. The Appendix is just a 1 paragraph generic description of the different dimensions and claim of unified training of the evaluation experts.
Need examples to illustrate what the factors mean and copy of what training materials or rubrics were given to evaluators with assessment of consistency.

Added a better description of how teh tool was used in a clinical workflow as a seperate service with copy-paste-edit into radiology reports.

Revised descriptions to acknowledge that even senior physician ratings cannot be relied upon as "gold standards" given the potential ambiguity in diagnostic interpretation.

Acknowledged ethical concerns before prospectively deploying an "AI-alone" study arm. Worthy of future consideration as it would be a much more compelling study result, and would still have a senior physician human-in-the-loop, so not a completely autonomous diagnostic system.

Overall, my prior suggestions have largely been addressed, but there are still a couple elements that would benefit from further clarification or description.

(Remarks on code availability)

Reviewer #4

(Remarks to the Author)

The authors have addressed some of the previous comments. However, there remain concerns regarding the lack of rigorous prospective evaluation (which is the main motivation of the study) and the potentially overstated clinical benefits of using an AI model for report generation.

Major Points:

1) Study design and focus
Most of the results presented in the manuscript are retrospective; only the first study is prospective. I recommend focusing the manuscript on the prospective study, as it aligns with the stated objective. The retrospective studies could be moved to a separate manuscript, as they involve different datasets, outcomes, and analyses, which currently creates confusion.

2) Clinical relevance of outcomes
While several outcomes are statistically significant, the magnitude of these differences does not appear clinically meaningful. This applies to quality scores, agreement scores, and reading times (see detailed comments below).

3) Interpretation and language
As noted in my first review, the language used to interpret the results is overly confident (e.g., “notably,” “exceptional ability”). I recommend revising the manuscript to present conclusions in proportion to the observed results, which, based on the data, do not demonstrate exceptional clinical applicability.

Specific Comments:

Results

Report quality scores: Are these reported as integers? For example, a mean difference between 4.36 and 4.12 is statistically significant but seems unlikely to be clinically meaningful.

Agreement scores: Similarly, a difference between 4.30 and 4.14 may not be clinically relevant despite statistical significance.

Reading time: A mean difference of 27 seconds appears clinically minor, especially since there was no change for complex cases, which typically require more time for radiologists without AI assistance.

Retrospective evaluation: The rationale for including this analysis is unclear. This likely belongs to a separate study or manuscript.

“Performance of Janus-Pro-CXR”: It is unclear whether these results are from the prospective or retrospective evaluations, or something else entirely. Which dataset was used?

“Validation of Multi-Image Input Capability of the Model”: The need for this additional analysis is not clear, especially given the multiple other analyses already presented.

Figures and Tables

Table 1 still reports retrospective evaluation. Consider removing it. Additionally, the p-value row is unclear; p-values should be reported as a separate column rather than a row.

Figure 1: Panels D and E have identical axes labels, making it difficult to distinguish between them. At a minimum, each panel should include a descriptive title.

(Remarks on code availability)

Version 1:

Reviewer comments:

Reviewer #1

(Remarks to the Author)
I thank the authors for their revised manuscript. The study adapts and tests a lightweight multimodal large language model based on Janus-Pro (Janus-Pro-CXR) fully fine-tuned in a multi-step process on MIMIC-CXR and CheXpert Plus and a novel multicenter dataset (CXR-27), for its capability to create CXR reports. The model is evaluated in a retrospective and in a prospective setting.

The authors have addressed my previous remaining feedback and suggestions (regarding unclear statements and

suboptimal case selection) adequately. I have no further concerns at this time.

(Remarks on code availability)
I have not tried to run the code, but I read it and it has not changed since the last submission; as it largely builds on DeepSeeks repositories it is expected to be executable. The associated GitHub repository holds the code and instructions for inference (specifically, generating a report based on a provided CXR image). Training code is not provided, but will be made available after publication as per the author's comments.

Reviewer #2

(Remarks to the Author)
I thank the authors for their thorough responses to my previous comments as well as comment from the other reviewers. The manuscript appears to have been strengthened on multiple fronts, including but not limited to the discussion section that now includes expanded discussion of the choice of models (generalist vs specialist). The updated supplementary appendices including the expert panel training protocol and other minor updates to the clinical impact aspects of the manuscript appear to be well done. In my re-reading of the manuscript, I noted some of the study strengths including the emphasis on the prospective aspects as well as clinical impact. All in all, the authors addressed the comments and concerns I had. I have no further revision request.

(Remarks on code availability)
In my previous iteration of review, I have reviewed the code at a higher level including the readme and deemed it okay, although I did not compile the code myself to verify that it runs successfully.

Reviewer #3

(Remarks to the Author)
The responses to my prior review comments appear to be appropriate with further discussion on tradeoffs between generalist vs. specific models, and better methodologic description of the human evaluation process.

I have no other major revision comments to add.

(Remarks on code availability)

Reviewer #4

(Remarks to the Author)
Most of my major concerns have been mainly addressed, while remaining minor concerns have been already addressed by the other Reviewers.

Remaining notes:
Line 125: “significantly” should be “statistically significant”.
Line 128: remove “notably”
Line 136-139: it’s an unspecified subgroup analysis, P-values are not needed, only report confidence intervals.
Add “in the retrospective study” to the title of results section “Second outcomes” (line 140) (if it only reports results from retrospective analysis).

(Remarks on code availability)

Open Access This Peer Review File is licensed under a Creative Commons Attribution 4.0 International License, which permits use, sharing, adaptation, distribution and reproduction in any medium or format, as long as you give appropriate credit to the original author(s) and the source, provide a link to the Creative Commons license, and indicate if changes were made.
In cases where reviewers are anonymous, credit should be given to 'Anonymous Referee' and the source.
The images or other third party material in this Peer Review File are included in the article’s Creative Commons license, unless indicated otherwise in a credit line to the material. If material is not included in the article’s Creative Commons license and your intended use is not permitted by statutory regulation or exceeds the permitted use, you will need to obtain permission directly from the copyright holder.
To view a copy of this license, visit https://creativecommons.org/licenses/by/4.0/

Responses to the reviewers' comments

On behalf of all my co-authors, I thank the editor and all reviewers for their constructive comments and suggestions on the revised manuscript, which greatly help us improve the quality of this work. We have substantially revised the manuscript in accordance with the reviewers' suggestions, carefully examined the entire manuscript, and made corresponding corrections. We have addressed all comments in a point-by-point format, and the revisions have been highlighted in red.

Sincerely,

Huangxuan Zhao

REVIEWER COMMENTS

Reviewer #1 (Remarks to the Author):

I thank the authors for their revised manuscript presenting Janus-Pro-CXR, a lightweight multimodal large language model for chest X-ray (CXR) reporting.
The authors have addressed most of my previous feedback and suggestions -- especially pursuing full fine-tuning and adding support for a second image as input -- with considerable revisions and improved their manuscript. Please find a more detailed updated review and my lingering minor concerns below.
Response: Thank you sincerely for your meticulous review. We also appreciate your recognition of our efforts, including full model fine-tuning, support for multi-image input and improved methodology details. Your affirmation of the prospective study's strength and our rigorous data and statistical design means a lot to us. Thank you again for your valuable input.

Summary / Key Results (Updated)
The study adapts and tests a lightweight (1B parameters) multimodal large language model based on Janus-Pro, (now) fully fine-tuned in a multi-step process on MIMIC-CXR and CheXpert Plus (after revision) and a novel multicenter dataset (CXR-27), for its capability to create CXR reports. The model is evaluated in a retrospective and in a prospective setting.

A major strength of the study remains the prospective part, in which primary outcomes and results comparing AI-assisted reports vs. unassisted reports (after revision) were
- Improved report quality scores of AI-assisted reports (4.36 ± 0.50 vs. 4.12 ± 0.80 (higher is better, $P < 0.001$),
- Higher pairwise preference of AI-assisted reports (preference by ≥3 out of 5 readers in 54.3%),
- Higher agreement with AI-assisted reports (RADPEER score 4.30 ± 0.57 vs. 4.14 ± 0.84

(higher is better, $P < 0.001$)), and
- Shorter reading time (reduction of 27s (-18.3%), $P < 0.001$), 32.5s/-16.4% for complexer cases with >= 3 findings).

In the retrospective study, automated report generation metrics and report quality scores were assessed and the model compared favorably to comparably sized and slightly bigger multimodal report generation models and general-domain models.

Revision
In the current revision, the authors have enhanced their earlier manuscript by
- Adapting the model to accommodate two radiograph inputs (for including a prior study or a second view). This is much closer to the needs of clinical radiology and the authors find the additional incorporation of lateral views to yield significantly better performance.
- Perform full fine-tuning instead of only parameter-efficient fine-tuning with LoRA, yielding higher performance (see updated key results above)
- Adding comparisons with other recent CXR reporting models
- Adding Chexpert Plus to their training dataset
- Adding fine-tuning details, details on dataset cleaning, adding details how confidence intervals were constructed

Originality and significance (Updated)
CXR is the most widely addressed imaging modality for AI-based report generation. Many studies have used automatic metrics for radiology report generation, and a few studies have found a beneficial effect of AI-generated CXR reports on reading time (e.g., Chen et al. CheXagent https://arxiv.org/abs/2401.12208 , Hong et al., https://doi.org/10.1148/radiol.241646 , Hong et al. https://doi.org/10.1016/j.jacr.2025.09.015).

The present manuscript is still one of the first to present a randomized, single-blinded prospective approach. Huang et al. (https://jamanetwork.com/journals/jamanetworkopen/fullarticle/2834943) recently published a study that prospectively evaluated a CXR reporting model and found a 15.5% reduction in reporting time, in line with the observed effects presented here; however, Huang et al. did not publish their model.

Reporting time reduction is a key metric for potential adoption of radiology AI systems with real-world impact, and as such, the study will be of interest to many in radiology as well as to other specialties that use lightweight, domain-adapted, vision-grounded language models for report/note generation.

Data & methodology (Updated)
Data and methodology of this study are appropriate and mostly allow for reproduction.
- The authors use public datasets (MIMIC-CXR and CheXpert Plus), a novel multi-center dataset (CXR-27) and a prospectively collected dataset from three hospitals. Dataset creation

incl. inclusion and exclusion criteria and dataset incl. patient characteristics are presented in detail in the flowchart and the supplementary materials. Previous errors in the flowcharts have been addressed.
- The model builds on a publicly available model (DeepSeek Janus-Pro).
- The associated GitHub repository holds the code and instructions for inference.
- The associated Huggingface repository holds the model weights for Janus-Pro-CXR-Zero and Janus-Pro-CXR-Final.
- (Updated) It has now been documented which DeepSeek model was used for label extraction & the prompts have been documented.

Use of statistics and treatment of uncertainties:
- Sample size calculation has been conducted using preliminary trial data.
- Statistical tests are appropriate.
- (Updated) Confidence intervals are reported, the revision also provided insight into their construction.
- (Updated) Error bars are present and have been explained more thoroughly through the revision.

Conclusions:
- The presented conclusions are supported by the results.

Suggested Improvements (Updated)

- (Updated) The presented model has now been fully fine-tuned and extended to enable more than one image input.
- (Updated) All previous minor suggestions have been addressed.
- (Updated) Training code is not provided, but the authors note that it will be made public after the article is accepted.
Q1. - (New) It's not clear what you mean by "with testing strictly conducted in accordance with the official division standards of this dataset" (Pg. 13, l. 333f)

Response: Thank you for your suggestion. Regarding the division of training and test sets for the MIMIC dataset, its official standards have been clearly specified in Table 3 on Page 4 of the paper (*MIMIC-CXR-JPG, A LARGE PUBLICLY AVAILABLE DATABASE OF LABELED CHEST RADIOGRAPHS*, https://arxiv.org/pdf/1901.07042). This study strictly adhered to this official division framework for conducting analyses; for instance, the 2,365 images used for testing in this study were all sourced from the officially defined test set mentioned above. We have revised the relevant ambiguous expressions in the revised manuscript and cited the aforementioned paper. Thank you again for your guidance!

Edema	26,455 (11.6%)	25,246.0 (11.1%)	11,781.0 (5.2%)	2,351 (1.0%)
Enlarged Cardiomediastinum	7,004 (3.1%)	5,271.0 (2.3%)	9,307.0 (4.1%)	255 (0.1%)
Fracture	3,768 (1.7%)	880.0 (0.4%)	299.0 (0.1%)	884 (0.4%)
Lung Lesion	6,129 (2.7%)	842.0 (0.4%)	1,020.0 (0.4%)	296 (0.1%)
Lung Opacity	50,916 (22.3%)	2,868.0 (1.3%)	2,110.0 (0.9%)	2,531 (1.1%)
No Finding	75,163 (33.0%)	-	-	3,906 (1.7%)
Pleural Effusion	53,188 (23.3%)	27,072.0 (11.9%)	5,345.0 (2.3%)	1,667 (0.7%)
Pleural Other	1,961 (0.9%)	120.0 (0.1%)	728.0 (0.3%)	93 (0.0%)
Pneumonia	15,769 (6.9%)	24,205.0 (10.6%)	17,789.0 (7.8%)	1,422 (0.6%)
Pneumothorax	9,317 (4.1%)	42,335.0 (18.6%)	868.0 (0.4%)	1,328 (0.6%)
Support Devices	65,637 (28.8%)	3,070.0 (1.3%)	96.0 (0.0%)	1,831 (0.8%)

releasing the test set. The validation set contains a random set of 500 patients and all of their associated studies. This set is made publicly available in a separate 'valid' folder. Finally, all remaining studies are made available in the training set. Table 3 provides summary information for studies in the three datasets. Note the enrichment of findings in the test set caused by the stratified sampling done to ensure sufficient coverage of all pathologies.

Table 3: Summary of the images split into training, validation, and test sets.

Dataset	Train	Validate	Test
Number of images	368960	2991	5159
Frontal	248020 (67.2%)	2041 (68.2%)	3653 (70.8%)
Lateral	120795 (32.7%)	949 (31.7%)	1502 (29.1%)
Other	145 (0.0%)	1 (0.0%)	4 (0.1%)
Number of studies	222758	1808	3269
with a finding	170420 (76.5%)	1394 (77.1%)	2912 (89.1%)
Number of patients	64586	500	293
with a finding	44157 (68.4%)	344 (68.8%)	288 (98.3%)

4

Page 15, Lines 389-392: Among these, 384,208 images were allocated to the first-stage model fine-tuning, and 2,365 images were designated as the test set. For MIMIC-CXR, testing was strictly conducted in accordance with its official train-test split standards[45]. Specifically, the 2,365 test images in this study were directly selected from the official test set defined in the dataset's core documentation.

Page 22, Lines 627-628: 45. Johnson, A.E.W., et al. MIMIC-CXR-JPG, a large publicly available database of labeled chest radiographs. arXiv:1901.07042 (2019).

Q2. - (New) The new Supplementary "Table" 6 shows another suboptimal performance (model calls the effusion reduced in comparison to prior, while in reality, it has slightly progressed), consider using another example or mention this is a failure example (similar to Supplementary "Table" 8).

Response: Thank you for your valuable suggestion. Supplementary Table 8 (previously numbered as Supplementary Table 6 prior to the addition of two new supplementary tables) presents an example where a prior chest X-ray and the current chest X-ray serves as model input. In light of your professional advice, we have replaced it with a more appropriate case to more accurately demonstrate the model's performance in this type of input scenario.

Supplementary Table 8 An example of historical chest radiographs and current chest radiographs as model inputs.

Chest radiographs	Reference report	AI-generated report
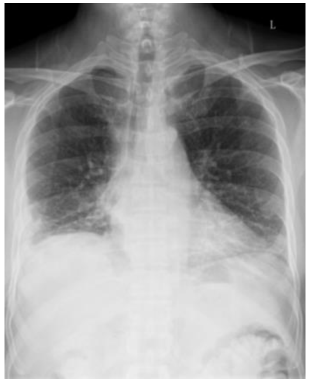<br>Current chest radiograph<br>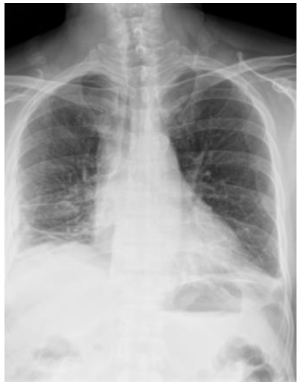<br>Historical chest radiograph	**Findings:** 1. Postoperative changes in the right lung with minimal residual pneumothorax/pleural air on the right side, showing improvement compared to previous findings. 2. Linear and patchy dense opacities in the lower lung fields bilaterally, more pronounced on the right, suggestive of inflammation/atelectasis, showing improvement compared to previous findings. 3. Minimal right pleural effusion; the left costophrenic angle appears largely clear. 4. The cardiac silhouette is slightly prominent.<br>**Impression:** Findings as described above.	**Findings:** 1. Postoperative changes in the right lung, with minimal pneumothorax on the right side and minimal subcutaneous emphysema in the right chest wall, with improvement compared to the previous examination. 2. Linear and patchy opacities in the lower lung fields bilaterally, suggestive of inflammatory changes/segmental atelectasis, showing improvement compared with the previous examination. 3. Slightly thickened pleura in the right lower lung field, with minimal pleural effusion. Follow-up or CT examination is recommended. 4. Slightly thickened and blurred lung markings in both lungs, with a few linear opacities in the lower lung fields bilaterally, possibly due to chronic inflammatory changes. 5. Slightly enlarged cardiac silhouette.<br>**Impression:** Findings as described above.

Reviewer #1 (Remarks on code availability):

I have not tried to run the code, but I read it; as it largely builds on DeepSeeks repositories it is expected to be executable. The associated GitHub repository holds the code and instructions for inference (specifically, generating a report based on a provided CXR image). Training code is not provided, but will be made available after publication as per the author's comments.

Model weights can be downloaded from Huggingface.

Reviewer #2 (Remarks to the Author):

This is a revised version of a manuscript that details how authors developed a DeepSeek-based system for automatic Chest X-ray interpretation. In response to multiple reviewer comments, they now describe an architecture of the model that includes multimodal inputs including lateral/historical images as well the patient’s clinical history. They have also addressed issues of unfair comparisons by including direct comparisons with MAIRA-2 and CheXagent and an experimental set-up Janus-Pro-CXR-Zero that was not fine-tuned on their internal CXR-27 test set. The revised manuscript also expanded design choices to the Discussion section and the benefits of such an open-source model for medical image interpretation. Overall, the revised manuscript addresses a majority of the core requests and concerns.
Response: Thank you sincerely for your meticulous review. We especially appreciate your recognition of the study’s prospective trial design, focus on AI collaboration and advantages of the lightweight open-source model. Your acknowledgment that we have addressed most core requests is deeply valued. We appreciate your support once more.

Several notable strengths at this point include:
1. A prospective trial of an open-source chest-X-ray system to aid in augmenting clinical workflows.
2. The prospective trial’s design of measuring resident+AI collaboration demonstrates a more plausible synergy and workflow optimization rather than directly pitting AI and attending radiologists as done in previous work.
3. Lightweight model architecture caters to multimodal input and clinical history, which outperforms and/or is competitive with other state-of-the-art models, offering flexibility for hospitals with limited access to GPU resources or private on-premise usage. These are also open-sourced.

Several remaining issues that I request the authors to revise or address or acknowledge as limitation as appropriate include:
Q1. 1. The new architecture that integrates multimodal inputs was not tested on the prospective clinical trial which limits the generalizability of claims regarding this new architecture. It would be necessary to prospectively test this model or reword and restructure the manuscript to reflect this methodical design.

Response: Thank you for your valuable comments. First, targeting the common multi-image input scenario in clinical practice, namely posteroanterior (PA) and lateral chest radiographs, we supplemented prospective validation data from 50 patients (Supplementary Figure 3). This type of scenario is a core application scenario for the model's multimodal input function, and the relevant validation results help partially reflect the model's performance under multi-image input. We have supplemented these prospective validation results in the Results section (*Page 8, Lines 198-204*).

Meanwhile, we have not yet completed the full prospective validation of the model's longitudinal integration capability for historical chest radiographs. The main reason is that validating this capability relies on cases with complete sequences of historical chest radiographs, and complete imaging data for such cases can only be obtained through long-term clinical follow-up. Due to the relatively long recruitment cycle, it is difficult to establish a complete study cohort in the short term, which in turn results in a lack of sufficient cases to support full validation in this area at present. We have provided an explanation of this in the Limitations section (*Page 12, Lines 313-323*).

In future work, we will continue accumulating patient cohorts with historical images to gradually complete full prospective validation of the multi-image input architecture and further enhance the generalizability of the study's conclusions.

Page 8, Lines 198-204: In addition, we have conducted prospective validation involving 50 patients who had posteroanterior and lateral chest X-rays (Supplementary Figure 3). Specific results demonstrated that the AI-assisted group achieved a higher report quality score than the standard care group (4.37±0.50 vs. 4.16±0.76, P<0.001); the AI-assisted group also reached a higher agreement score than the standard care group (4.38±0.58 vs. 4.22±0.90, P<0.001); and in terms of work efficiency, the average report reading time for the AI-assisted group was shorter than the standard care group (120.5±46.9 seconds vs. 153.6±57.0 seconds, P<0.001).

Page 12, Lines 313-323: Second, the real-world applicability of multi-image integration capability is one of the core prerequisites for the successful clinical translation of multimodal medical imaging AI[38]. Currently, Janus-Pro-CXR has completed validation for multi-image input of posteroanterior and lateral chest radiographs, the most common scenario in clinical practice. Its performance advantages have been confirmed through prospective studies. However, complete prospective validation is still lacking for the longitudinal integration capability of historical chest radiographs. This capability refers to the analysis of dynamic changes in serial follow-up images. This is mainly because cases with complete sequences of historical chest radiographs can only be obtained through long-term clinical follow-up, resulting in a relatively long accumulation cycle. It is difficult to establish a complete cohort in the short term. Subsequent research will focus on advancing the prospective validation of this function to enhance the model's clinical applicability.

Page 18, Lines 482-487: ***Validation of Multi-Image Input Capability of the Model***

To verify the model's multi-image input capability, an additional 50 patients with both posteroanterior and lateral chest radiographs, as well as 50 patients with historical chest radiographs, were included in the retrospective study for evaluation. In the prospective study, another 50 patients with posteroanterior and lateral chest radiographs were enrolled for validation. The evaluation methods included report quality scores, agreement scores, and reading time.

Supplementary Figure 3 Prospective Validation for Multi-Image Input Scenarios Based on 50 Patients with Posteroanterior and Lateral Chest X-rays.

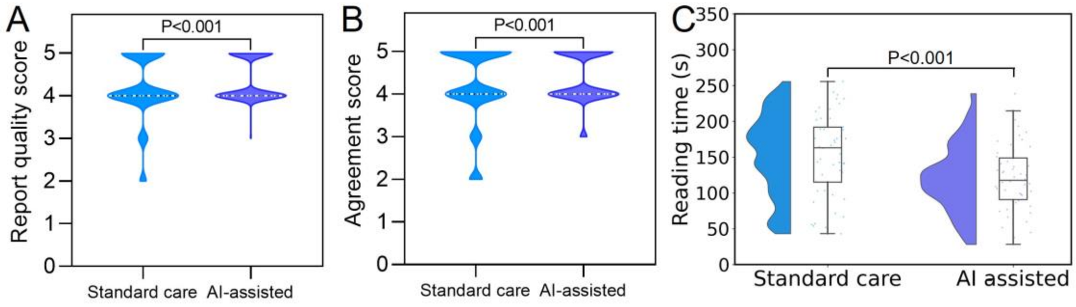

(A) Report quality scores for the standard care group and the AI assisted group in the prospective study (n=50). (B) Report agreement scores for the standard care group and the AI assisted group in the prospective study (n=50). The paired t-tests were used in (A) and (B). Each violin's width reflects data point density at respective values; white dashed lines show the median, black dashed lines the interquartile range (25th–75th percentiles). (C) Reading time for the standard care group and the AI assisted group in the prospective study (n=50). The paired t-test was used and the center line corresponds to the median, and the box is delineated by the first and third quartiles.

Q2. 2. The authors did not include a research setting that immediately sent the AI-generated reports for attending review to benchmark against the AI-generated reports vetted by residents. Without this research setting, the authors cannot claim true diagnostic reliability and workflow efficiency.

Response: Thank you for your suggestion. To address the limitation you identified, we first incorporated additional subjective assessments for AI generated reports in the prospective trial. This measure remedied the absence of evaluation for the specific scenario where "AI-generated reports are directly submitted to senior radiologists for review," thereby indirectly refining the comparative analysis of this workflow. As shown in Figures 3A and 3B, the data clearly demonstrate that the report quality scores of AI-generated group (3.18 ± 1.20) are lower than the corresponding scores of both the AI-assisted group (4.36 ± 0.50) and the standard care group (4.12 ± 0.80); the agreement scores of AI-generated reports (3.16 ± 1.19) are also lower than the AI-assisted group (4.30 ± 0.57) and the standard care group (4.14 ± 0.84).

Second, one of the primary endpoints of this study centers on AI's contribution to enhancing junior radiologists' work efficiency (e.g., reading time), and the review time of senior radiologists was not included in the evaluation scope. Combined with findings from the retrospective study, the accuracy of AI-generated reports when generated independently remains limited. Directly submitting AI-generated reports to senior radiologists without prior revision by junior radiologists would likely impose an additional workload on the former (e.g., extending their review time). Notably, the evaluation of efficiency in such senior radiologists' review procedures was not within the predefined scope of this study.

Finally, in future research, we will explicitly include a dedicated cohort for the scenario where "AI-generated reports are directly submitted to senior radiologists for review." This will enable us to systematically evaluate AI's utility across the full workflow of radiology report generation, with special emphasis on validating its impact on the efficiency of senior radiologists' review procedures. Once again, thank you for your guidance.

Page 13, Lines 333-341: On the other hand, although Janus-Pro-CXR outperforms Janus-Pro and GPT-4o in independent report generation in retrospective analyses, it still has significant limitations. This is further confirmed by its performance in independent report generation in the prospective study. It indicates that Janus-Pro-CXR can only serve as an auxiliary tool for radiologists at the current stage and cannot replace them. In future research, we will further optimize Janus-Pro-CXR's independent report generation capability. We will also explore the workflow model where AI-generated reports are directly submitted to senior physicians for review. This aims to provide more reliable evidence-based support for improving the efficiency of clinical translation.

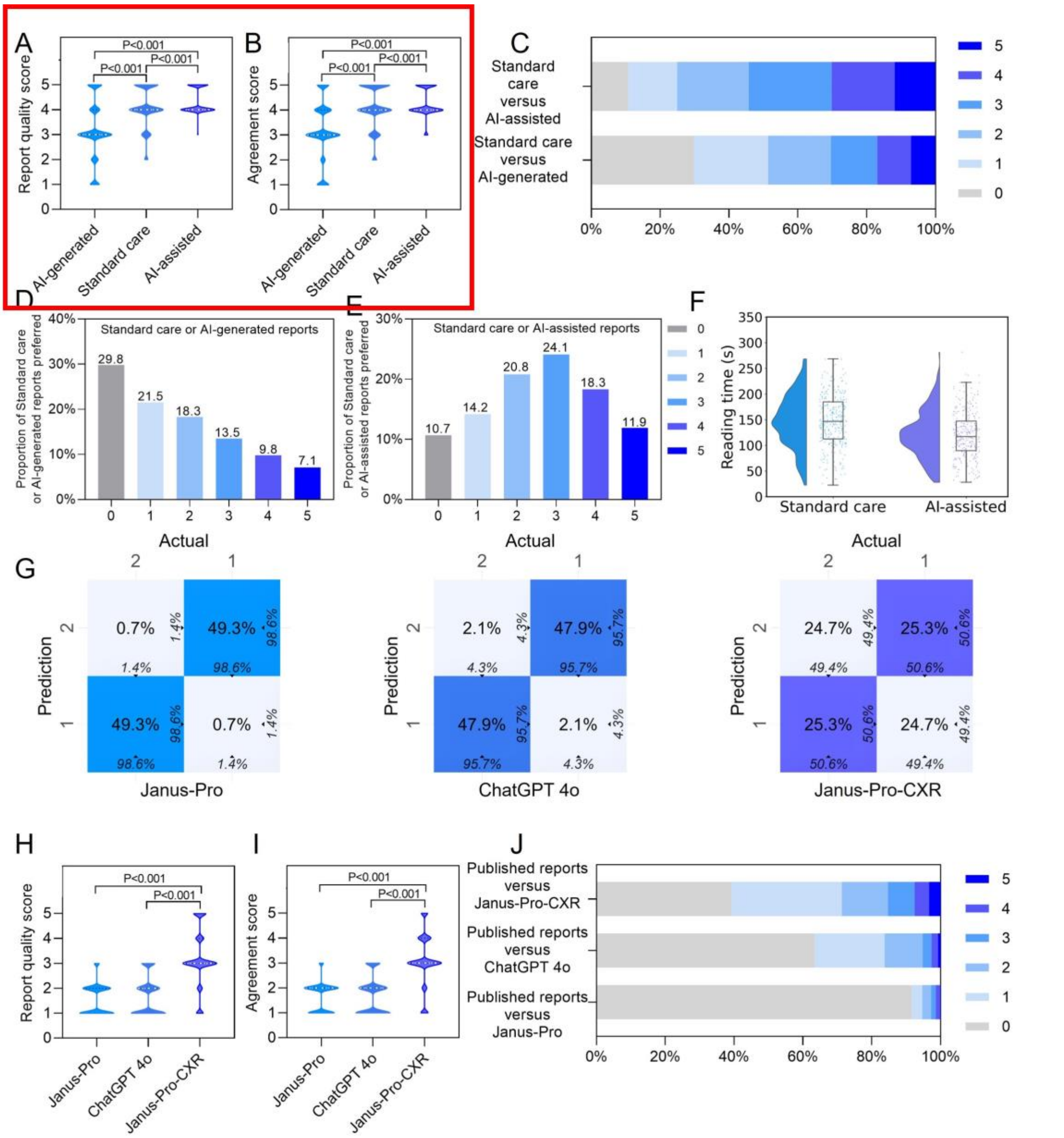

Figure 3. *Subjective evaluations of the prospective and retrospective studies. (A) Report quality scores for the AI-generated group, the standard care group, and the AI-assisted group in the prospective study (n=296). (B) Report agreement scores for the AI-generated group, the standard care group, and the AI assisted group in the prospective study (n=296). Repeated measures ANOVA determined statistical significance for (A) and (B). Each violin's width reflects data point density at respective values; white dashed lines show the median, black dashed lines the interquartile range (25th–75th percentiles). (C) Preference tests comparing reports generated by junior radiologists and those generated by AI, as well as those generated by junior radiologists with AI collaboration, in the prospective study. (D) Preference test comparing reports generated by junior radiologists and AI. (E) Preference test comparing reports generated by junior radiologists with AI collaboration. (F) Reading time for the standard care group and the AI assisted group in the prospective study (n=296). The paired t-test was used and the center line corresponds to the median, and the box is delineated by the first and third quartiles. (G) Confusion matrix for evaluators to identify reports generated by*

large models in the retrospective study. (H) Report quality scores of the three multimodal large language models in the retrospective study (n=300). (I) Report agreement scores of the three multimodal large language models in the retrospective study (n=300). Repeated measures ANOVA determined statistical significance for (H) and (I). Each violin's width reflects data point density at respective values; white dashed lines show the median, black dashed lines the interquartile range (25th–75th percentiles). (J) Preference tests comparing published reports with those generated by Janus-Pro-CXR, ChatGPT 4o, and Janus-Pro in the retrospective study.

Reviewer #2 (Remarks on code availability):

Based on my cursory review of the code (without having compiled it myself), the Readme content is adequate and the code appears to be available for download and execution. Assuming the code works as stated in the readme file, the authors have provided sufficient information to reproduce the model.

Reviewer #3 (Remarks to the Author):

I provided a mostly positive prior review of an earlier version of this manuscript, thus I focus my discussion below just on the responses to my prior review comments:
Response: Thank you for your continued support. We also appreciate your recognition of our revisions, such as expanding the physician reviewer sample size, re-testing the CXR-27 dataset and refining descriptions of clinical workflows. Your affirmation of the study's rigor is greatly appreciated. Thank you once again for your consistent guidance.

Appears study has effectively expanded physician reviewer sample size from 6 to 20 physicians to better account for clustered statistical power.

They redid a set of experiments on the CXR-27 dataset as there was some unusually poor performance of the CheXPert baseline on labeling. No clear reason why, but speculation of regional differences in clincial standards and linguistic conventions.

Q1. Added specific comparison against CXR specific models (e.g., MAIRA-2, CheXagent, ...)
Then argues towards more useful comparison to generalist models that are more available.
Then argues that clinical specialist systems is an important paradigm shift.

Can't have it both ways. Should add to discussion or limitations on how this specialist model may be good compared to generalist models, but that other specialist models could be as good but less available.

Response: Thank you for your suggestion. We have elaborated in detail on the content related to comparisons with other models in the "Discussion" section, and supplemented the reasons why the prospective study only compared with generalist models in the "Limitations" section.

Generalist models such as ChatGPT 4o exhibit remarkable strengths in versatility across diverse domains and robust natural language comprehension capabilities, and they have even shown preliminary potential in providing basic support for medical text processing. Yet when it comes to the specialized task of chest X-ray interpretation, Janus-Pro-CXR—a chest X-ray-specific model—holds core advantages over such generalist models, which primarily lie in two aspects: clinical adaptability and practical deployment value. First, it demonstrates superior detection accuracy for clinical signs of chest diseases. Second, in terms of terminology use, Janus-Pro-CXR strictly adheres to radiological diagnostic standards, effectively avoiding issues common in generalist models such as colloquial terminology and disorganized content structure. Third, Janus-Pro-CXR has a significantly lower deployment barrier: its lightweight architectural design eliminates the need for high-end hardware, enabling fast inference on standard workstations. This not only reduces reliance on cloud-based inference but also mitigates the risk of data privacy breaches.

On the other hand, existing chest X-ray-specific models, such as MAIRA-2 and CheXagent, have demonstrated considerable value in the field. For instance, they have achieved notable progress in key tasks, including the accurate detection of specific lesions and the clinical relevance of report content, and this has accumulated valuable experience for the clinical translation of chest imaging AI technologies. Nevertheless, when compared with these specialized models, Janus-Pro-CXR still maintains advantages in objective metrics related to report generation performance and key lesion detection capabilities. Furthermore, other chest X-ray-specific models generally have significant accessibility limitations. For instance, some do not open-source their model weights or training codes, which makes independent deployment difficult for clinical institutions. Others require high GPU memory that standard workstation configurations cannot support. This is precisely why we did not conduct comparisons with these specialist models in our prospective study, even though they may also exhibit equally good performance in certain tasks.

Page 11, Lines 273-293: Generalist models like ChatGPT 4o excel in cross-domain versatility and strong natural language comprehension, with preliminary potential in medical text processing support[3]. As a chest X-ray-specific model, Janus-Pro-CXR's core advantages over generalist models such as ChatGPT 4o lie primarily in two aspects: clinical adaptability and practical deployment value. First, it demonstrates better detection accuracy for clinical signs of chest diseases. Second, in terms of terminology use, Janus-Pro-CXR strictly adheres to radiological diagnostic standards, effectively avoiding issues common in generalist models such as colloquial terminology and disorganized content structure. Third, Janus-Pro-CXR has a significantly lower deployment barrier: its lightweight architectural design eliminates the need for high-end hardware, enabling fast inference on standard workstations. This not only reduces reliance on cloud-based inference but also mitigates the risk of data privacy breaches. On the other hand, existing chest X-ray-specific models (e.g., MAIRA-2[34], CheXagent[35]) have shown great field value, with progress in key tasks and valuable experience for chest imaging

AI's clinical translation. When compared with other chest X-ray-specific models, Janus-Pro-CXR achieves better performance in objective metrics related to report generation and key lesion detection. Furthermore, other chest X-ray-specific models generally have significant accessibility limitations[12,36]. For instance, some do not open-source their model weights or training codes, which makes independent deployment difficult for clinical institutions. Others require high GPU memory that standard workstation configurations cannot support. This is precisely why we did not conduct comparisons with these specialist models in our prospective study, even though they may also exhibit equally good performance in certain tasks.

Pages 12-13, Lines 324-330: Furthermore, this study still has certain limitations in aspects such as prospective clinical validation. Compared with other chest X-ray-specific models such as MAIRA-2[34] and CheXagent[35], Janus-Pro-CXR performs better in objective metrics related to report generation and key lesion detection. However, other specialized models generally have significant accessibility limitations. They may not be open-source or may require high GPU memory that standard configurations cannot support[12,36]. For this reason, we did not compare Janus-Pro-CXR with these models in the prospective study. Some of these models may still perform equally well in specific tasks.

Q2. Added a Supplementary Appendex 7 to better explain how the expert panel reviewed diagnostic reports along multiple dimensions (professionalism, logical coherence, diagnostic suggestions, and other report aspects).

This response is inadequate. The Appendix is just a 1 paragraph generic description of the different dimensions and claim of unified training of the evaluation experts.
Need examples to illustrate what the factors mean and copy of what training materials or rubrics were given to evaluators with assessment of consistency.
Response： Thank you for your valuable suggestions. We have supplemented the description of the training protocol and inter-rater reliability verification results in Supplementary Appendix 7. Meanwhile, we have provided the inter-rater reliability verification results for both retrospective and prospective assessments in the main text. Furthermore, we have added the scoring criteria and corresponding examples in Supplementary Appendix 8.

Supplementary Appendix 7 Standardized Training and Assessment for Subjective Evaluation

To ensure that the 5 radiologists participating in the subjective evaluation (all with 8-15 years of professional experience) have a consistent understanding of the standards for each evaluation dimension, standardized training was conducted prior to the evaluation. The total training duration was 3 hours, specifically including 1 hour of interpretation of evaluation criteria (including scoring criteria and examples) and 2 hours of group case practice. During the practice, the radiologists performed report quality scoring and consistency scoring (on a 1-5 scale) for 10 AI-generated reports and 10 clinically published reports, and discussed scoring differences to unify the standards. All evaluators participated in the entire training process and passed the post-training assessment to ensure they possessed unified evaluation competence.

The assessment scheme is as follows: Each of the 5 evaluators rated 20 reports (10 AI generated reports and 10 clinically published reports, randomly shuffled), and the Kendall's W coefficient was calculated. The strength of agreement between the observers was classified as poor (W＜0.2), fair (0.2 ≤W＜0.4), moderate (0.4 ≤W＜0.6), strong (0.6 ≤W＜0.8), or super (0.8 ≤W＜1.0).

The assessment results are as follows:

	Kendall's W	P value
Report Quality Score	0.871	<0.001
Agreement Score	0.845	<0.001

Supplementary Appendix 8 Radiology report quality scoring criteria (5-point likert scale).

Score	Criteria	Example
5	Flawless structure, terminology, and diagnostic reasoning; All findings precisely described with clinical context; Diagnosis matches gold standard perfectly	FINDINGS: The thoracic cage is bilaterally symmetrical. Bilateral lung fields show increased, disorganized interstitial markings; patchy increased density with blurred margins is seen in the lower lung fields. Bilateral diaphragmatic surfaces are smooth; costophrenic angles are slightly blunted. The cardiac silhouette is normal in size and shape.<br>IMPRESSION: Bilateral lower lung field exudative changes; Possible minimal bilateral pleural effusion or pleural thickening; Recommend short-term follow-up chest X-ray to assess lesion resolution.
4	Minor formatting issues or slight terminology deviations; 1-2 non-critical findings described less precisely; Diagnosis correctly but may lack nuance	FINDINGS: The thoracic cage is bilaterally symmetrical. Bilateral lung markings are increased and slightly disorganized; patchy areas of increased density are seen in the bilateral lower lung fields, with somewhat ill-defined margins. Bilateral diaphragmatic surfaces are smooth, and the costophrenic angles are slightly blunted. The cardiac silhouette is roughly normal in size and shape.<br>IMPRESSION: Bilateral lower lung field exudative changes; possible minimal bilateral pleural effusion; clinical correlation is recommended.
3	Basic sections present (findings/impression); Some findings unclear/partially wrong; The diagnostic direction is unclear or has deviations	FINDINGS: The thoracic cage is generally normal. Bilateral lung markings are generally unremarkable. Bilateral diaphragmatic surfaces are relatively smooth, and the costophrenic angles appear slightly blunted. The cardiac silhouette is generally normal in size.<br>IMPRESSION: Possible bilateral pleural effusion; Further clarification of the diagnosis is required.
2	Missing major sections or confusing flow; Multiple errors affecting interpretation; Diagnosis questionable	FINDINGS: The thoracic cage shows no obvious abnormalities. Small patchy high-density shadows are noted in both lungs (with unclear nature). Diaphragms were not carefully evaluated; the cardiac silhouette appears slightly enlarged?<br>IMPRESSION: Possible abnormalities in both lungs; Diaphragms were not carefully evaluated; The cardiac silhouette is enlarged.

1	Unreadable or medically dangerous errors; Critical findings missed/misrepresented; Diagnosis contradicts imaging	The thoracic cage is bilaterally symmetrical. Bilateral lung markings are markedly increased and disorganized. An extensive area of suspected consolidation is noted in the left lobe of the lung.

Added a better description of how teh tool was used in a clinical workflow as a seperate service with copy-paste-edit into radiology reports.

Revised descriptions to acknowledge that even senior physician ratings cannot be relied upon as "gold standards" given the potential ambiguity in diagnostic interpretation.

Acknowledged ethical concerns before prospectively deploying an "AI-alone" study arm. Worthy of future consideration as it would be a much more compelling study result, and would still have a senior physician human-in-the-loop, so not a completely autonomous diagnostic system.

Overall, my prior suggestions have largely been addressed, but there are still a couple elements that would benefit from further clarification or description.

Reviewer #4 (Remarks to the Author):

The authors have addressed some of the previous comments. However, there remain concerns regarding the lack of rigorous prospective evaluation (which is the main motivation of the study) and the potentially overstated clinical benefits of using an AI model for report generation.

Response: Thank you sincerely for your meticulous feedback and guidance. We appreciate your recognition of our initial efforts and have made targeted revisions as you suggested. We prioritized prospective results and clarified that core conclusions rely on them, with retrospective data serving as support. We added explanations for the clinical relevance of outcomes, softened overly confident language, and optimized tables and figures to enhance clarity. Thank you again for your input, which has strengthened the manuscript's rigor and relevance.

Major Points:

Q1. 1) Study design and focus

Most of the results presented in the manuscript are retrospective; only the first study is prospective. I recommend focusing the manuscript on the prospective study, as it aligns with the stated objective. The retrospective studies could be moved to a separate manuscript, as they involve different datasets, outcomes, and analyses, which currently creates confusion.

Response: Thank you for your valuable suggestions on the logical presentation of the study.

Following your suggestions, we have organized and optimized the study's logical framework to enhance the clarity of expression. We have prioritized the reporting of prospective results in the Results section, and centered the Discussion section around these prospective findings. We have explicitly stated in the "Results" and "Discussion" sections that " the core conclusions of this study are grounded in the findings of the prospective study, while the retrospective study merely provides methodological scaffolding and supporting findings," which further emphasizes the primary-secondary relationship and eliminates potential confusion.

On the other hand, considering that retrospective validation results provide methodological support for the prospective study, facilitating the understanding of its design rationale and result reliability, we have retained the retrospective data. This is consistent with the research ideas and designs of previous studies (*Nat Med. 2025 Jul;31(7):2255-2268. doi: 10.1038/s41591-025-03724-5.; Lancet Digit Health. 2024 Apr;6(4):e261-e271. doi: 10.1016/S2589-7500(23)00268-6.*). Finally, it should be noted that if the retrospective section is removed, it will be impossible to respond to the relevant questions raised by other reviewers, and thus the revision process will be difficult to proceed with. We will proactively adopt and implement your suggestions in future studies. Thank you again for your guidance!

Page 5, Lines 109-112: Baseline patient data are provided in Supplementary Tables 1 and 2. The core conclusions of this study are grounded in the findings of the prospective study, while the retrospective study merely provides methodological scaffolding and supporting findings.

Page 8, Lines 206-209: In this study, Janus-Pro-CXR, an intelligent report generation system tailored for radiology clinical practice, was developed based on the DeepSeek multimodal model (Janus-Pro). The core conclusions of this study are grounded in the findings of the prospective study, while the retrospective study merely provides methodological scaffolding and supporting findings.

Q2. 2) Clinical relevance of outcomes
While several outcomes are statistically significant, the magnitude of these differences does not appear clinically meaningful. This applies to quality scores, agreement scores, and reading times (see detailed comments below).
Response: Thank you for your valuable suggestions. Regarding the issues you raised concerning the scoring format and reading time, we provide the following detailed explanations.

First, regarding the scoring format, the raw scores for both report quality and agreement are integers. Each evaluator independently assigns scores on a 1-5 scale, and the decimal values presented in the study represent the average of all evaluators' scores. This approach aims to more objectively reflect the overall scoring level and avoid the impact of individual evaluators' subjective biases on the interpretation of results. It is important to note that the improvement in subjective scores is not merely a numerical change, but directly linked to clinical practical value. The scoring method used in this study is consistent with that of previous studies (*Radiology. 2025 Mar;314(3):e241646. doi: 10.1148/radiol.241646.; Radiology. 2025 Mar;314(3):e241476. doi: 10.1148/radiol.241476.*) and the scoring criteria have been further refined. In real clinical settings, such improvements in scores can effectively reduce medical

risks arising from ambiguous report descriptions, unclear diagnostic logic, or missing key information. Especially for primary care institutions, more standardized report quality can significantly lower the misdiagnosis rate caused by information biases and provide a more reliable reference for clinical diagnosis and treatment decisions. After all, in medical practice, even minor biases in reports may impose a heavy medical burden on patients or even endanger their lives.

Second, we further address the clinical significance of reading time. Considering the characteristics of clinical practice in large-scale medical centers in China, radiologists can read approximately 200 chest radiographs per person in a single full workday if they dedicate their time exclusively to this task. Based on the 27 seconds saved per report in the AI-assisted group in this study, radiologists can accumulate a total of 90 minutes of savings per day. This time can be effectively allocated to two key clinical tasks. The first is conducting in-depth analysis of complex cases. The second is alleviating visual fatigue caused by prolonged and high-intensity image reading. This helps reduce the risk of missed diagnoses due to fatigue and indirectly improves the safety of diagnosis and treatment. Furthermore, regarding the complex case scenario you are concerned about, which is explicitly defined in this study as "cases with ≥3 imaging findings", our supplementary subgroup analysis results show that even in such scenarios with higher diagnostic difficulty, the AI-assisted group still demonstrates a significant time advantage with an average of 32.5 seconds saved per case. This result further confirms the practical value of the model in high-demand clinical scenarios, rather than being applicable only to simple cases.

We have added the above content in the Discussion section to more clearly elaborate on the connection between technological improvements and clinical value. We hope these supplementary explanations address your concerns, and we would like to thank you again for your meticulous guidance!

Page 10, Lines 252-265: The results revealed that Janus-Pro-CXR significantly enhanced the diagnostic quality of junior radiologists, improving report quality scores by 0.25 points, while also reducing the average report time by 27.0 seconds (18.3% reduction). These improvements are highly aligned with core clinical needs. Specifically, enhanced report quality can directly reduce the incidence of missed diagnoses and misdiagnoses, thereby lowering risks in clinical decision-making. After all, in medical practice, even minor biases in reports may impose a heavy medical burden on patients or even endanger their lives. Meanwhile, shorter image interpretation time can optimize radiology workflows. Considering the characteristics of clinical practice in large-scale medical centers in China, the radiologists read approximately 200 chest radiographs per person in a day if they devote the entire day solely to this task. Based on the 27 seconds saved per report in the AI-assisted group in this study, radiologists can accumulate a total of 90 minutes of savings per day. This saved time can be used not only for in-depth analysis of complex cases but also for alleviating visual fatigue caused by prolonged and high-intensity image reading, thereby reducing the risk of missed diagnoses due to fatigue and indirectly improving the safety of diagnosis and treatment.

Q3. 3) Interpretation and language

As noted in my first review, the language used to interpret the results is overly confident (e.g., “notably,” “exceptional ability”). I recommend revising the manuscript to present conclusions in proportion to the observed results, which, based on the data, do not demonstrate exceptional clinical applicability.

Response: Thank you for your meticulous reminder. We agree with your comments and have made revisions strictly in accordance with your suggestions.

Pages 2-3, Lines 46-49: Our system outperforms state-of-the-art X-ray report generation models in automated report generation, surpassing even larger-scale models including ChatGPT 4o (200B parameters), while demonstrating reliable ~~robust~~ detection of six clinically critical radiographic findings (area under the curve, AUC > 0.8).

Page 6, Lines 155-157: The agreement scores between the Janus-Pro-CXR-generated reports and the published reports reached 3.10 ± 1.05, ~~notably~~ higher than the agreement scores for Janus-Pro (1.66 ± 0.60) and ChatGPT 4o (1.74 ± 0.75) (Figure 3I).

Page 7, Lines 165-167: Evaluation on the MIMIC-CXR test set highlighted the model's ~~exceptional~~ ability to achieve balanced performance in recognizing both common and rare diseases.

Page 7, Lines 169-170: ~~Notably~~ Additionally, the model’s Radiology Graph (RadGraph) F1 score for the medical entity relationship extraction task reached 25.8.

Page 7, Lines 173-174: ~~Notably~~ Additionally, Janus-Pro-CXR-Zero also performed ~~exceptionally~~ well in the CXR-27 test set, ranking second in most indicators.

Page 8, Lines 209-212: The domain-specific optimization of Janus-Pro-CXR has yielded substantial advantages in natural language generation metrics and demonstrated ~~outstanding~~ good performance in diagnostic accuracy and report generation quality.

Pages 8-9, Lines 212-214: Prospective clinical validation confirmed that Janus-Pro-CXR not only significantly enhances the diagnostic accuracy of radiologists but also effectively ~~markedly~~ improves their work efficiency.

Page 10, Lines 266-267: Furthermore, the positive rate of pneumonia diagnosis in the AI-assisted group was ~~notably~~ higher than in the standard care group (52.4% vs. 36.1%, P < 0.001).

Specific Comments:

Results

Q4. Report quality scores: Are these reported as integers? For example, a mean difference between 4.36 and 4.12 is statistically significant but seems unlikely to be clinically meaningful.

Response: Thank you for your kind suggestion. With regard to this matter, we have already

addressed the concerns you raised previously, and we would like to kindly direct you to Q2 for your reference.

Q5. Agreement scores: Similarly, a difference between 4.30 and 4.14 may not be clinically relevant despite statistical significance.

Response: Thank you for your kind suggestion. With regard to this matter, we have already addressed the concerns you raised previously, and we would like to kindly direct you to Q2 for your reference.

Q6. Reading time: A mean difference of 27 seconds appears clinically minor, especially since there was no change for complex cases, which typically require more time for radiologists without AI assistance.

Response: Thank you for your kind suggestion. With regard to this matter, we have already addressed the concerns you raised previously, and we would like to kindly direct you to Q2 for your reference.

Q7. Retrospective evaluation: The rationale for including this analysis is unclear. This likely belongs to a separate study or manuscript.

Response: Thank you for your kind suggestion. With regard to this matter, we have already addressed the concerns you raised previously, and we would like to kindly direct you to Q1 for your reference.

Q8. “Performance of Janus-Pro-CXR”: It is unclear whether these results are from the prospective or retrospective evaluations, or something else entirely. Which dataset was used?

Response: Thank you for your detailed suggestions. Regarding the relevant results of "Janus-Pro-CXR Performance" that you have focused on, it should be made clear that all results in this section are derived from retrospective evaluation, and we have revised the subheading to "Performance of Janus-Pro-CXR in the retrospective study". On the other hand, in accordance with your suggestions, we have supplemented the datasets used in the main text to ensure that data traceability and evaluation logic are clear at a glance.

Page 7, Line 160: ***Performance of Janus-Pro-CXR in the retrospective study***

Page 7, Lines 167-169: Our model ranks first among all models in terms of Micro-avg F1-5 (63.4) and Macro-avg F1-5 (55.1), and second in terms of Micro-avg F1-14 (59.9) and Macro-avg F1-14 (42.3) in the MIMIC-CXR test set.

Page 7, Lines 175-180: The model showed high consistency with the reference standard in classifying diseases such as support devices and pleural effusion in the CXR-27 test set (Figure 4D). Diagnostic performance was also strong (Figure 4E, Supplementary Figure 2, and Supplementary Table 7), with AUC values > 0.8 for six key findings, including support devices (AUC = 0.931), pleural effusion (AUC = 0.931), and pneumothorax (AUC = 0.921) in the CXR-27 test set.

Q9. “Validation of Multi-Image Input Capability of the Model”: The need for this additional analysis is not clear, especially given the multiple other analyses already presented.

Response: Thank you for your suggestion. The addition of this section of analysis is primarily based on the suggestions of other reviewers during the previous round of review. Given that radiologists in clinical practice sometimes need to integrate multiple chest images of the same patient (such as posteroanterior [PA] and lateral chest X-rays) to make comprehensive judgments on the patient's condition, rather than relying solely on a single image, it was suggested that we supplement the verification of the model's ability to process multi-image inputs.

Figures and Tables

Q10. Table 1 still reports retrospective evaluation. Consider removing it. Additionally, the p-value row is unclear; p-values should be reported as a separate column rather than a row.

Response: Thank you for your suggestion. We have revised Table 1 to highlight the key conclusions of the prospective study, presenting the P-values in a single column, while the redundant content has been placed in the supplementary information.

Table 1. Differences in primary outcome measures of the prospective study.

	Standard care	AI-assisted	P value
Report Quality Score	4.12±0.80	4.36±0.50	<0.001
Agreement Score	4.14±0.84	4.30±0.57	<0.001
Reading time	147.6±51.1	120.6±45.6	<0.001

Data were presented as mean ± SD where appropriate. Paired t-test was used to analyze the differences between the two groups (n=296).

Q11: Figure 1: Panels D and E have identical axes labels, making it difficult to distinguish between them. At a minimum, each panel should include a descriptive title.

Response: Thank you very much for your suggestion. Regarding Figure 1, there is no issue where the axis labels of Panels D and E are identical. We wonder if your question might have originated from Panels D and E in Figure 3. Admittedly, Panels D and E in Figure 3 do appear somewhat similar, so we have added descriptive titles to distinguish between them.

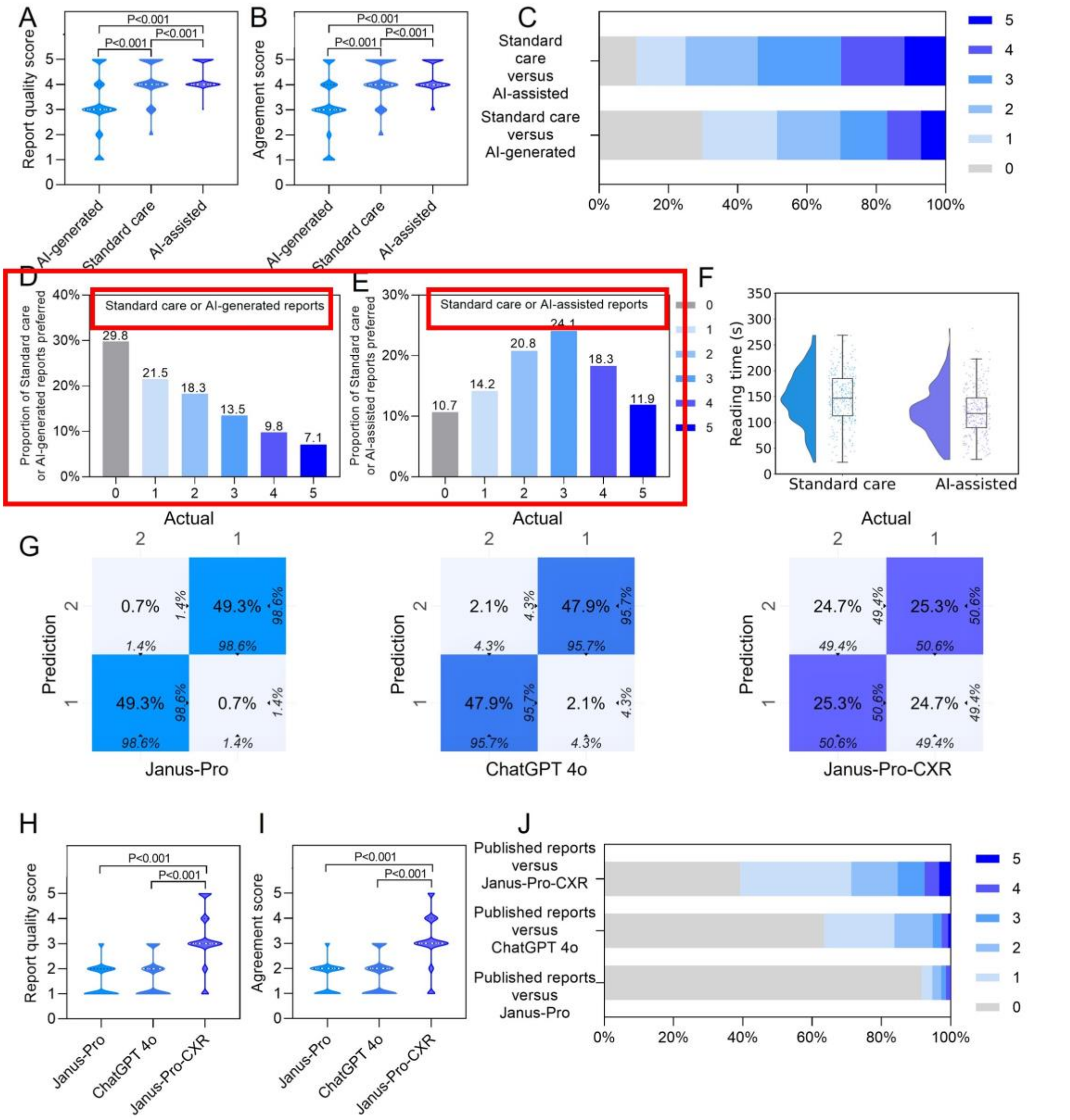

Figure 3. *Subjective evaluations of the prospective and retrospective studies. (A) Report quality scores for the AI-generated group, the standard care group, and the AI-assisted group in the prospective study (n=296). (B) Report agreement scores for the AI-generated group, the standard care group, and the AI assisted group in the prospective study (n=296). Repeated measures ANOVA determined statistical significance for (A) and (B). Each violin's width reflects data point density at respective values; white dashed lines show the median, black dashed lines the interquartile range (25th–75th percentiles). (C) Preference tests comparing reports generated by junior radiologists and those generated by AI, as well as those generated by junior radiologists with AI collaboration, in the prospective study. (D) Preference test comparing reports generated by junior radiologists and AI. (E) Preference test comparing reports generated by junior radiologists with AI collaboration. (F) Reading time for the standard care group and the AI assisted group in the prospective study (n=296). The paired t-test was used and the center line corresponds to the median, and the box is delineated by the first and third quartiles. (G) Confusion matrix for evaluators to identify reports generated by large models in the retrospective study. (H) Report quality scores of the three multimodal large*

language models in the retrospective study (n=300). (I) Report agreement scores of the three multimodal large language models in the retrospective study (n=300). Repeated measures ANOVA determined statistical significance for (H) and (I). Each violin's width reflects data point density at respective values; white dashed lines show the median, black dashed lines the interquartile range (25th–75th percentiles). (J) Preference tests comparing published reports with those generated by Janus-Pro-CXR, ChatGPT 4o, and Janus-Pro in the retrospective study.

Responses to the reviewers' comments

On behalf of all my co-authors, I thank the editor and all reviewers for their constructive comments and suggestions on the revised manuscript, which greatly help us improve the quality of this work. We have substantially revised the manuscript in accordance with the reviewers' suggestions, carefully examined the entire manuscript, and made corresponding corrections. We have addressed all comments in a point-by-point format, and the revisions have been highlighted in red.

Sincerely,

Huangxuan Zhao

REVIEWERS' COMMENTS

Reviewer #1 (Remarks to the Author):

I thank the authors for their revised manuscript. The study adapts and tests a lightweight multimodal large language model based on Janus-Pro (Janus-Pro-CXR) fully fine-tuned in a multi-step process on MIMIC-CXR and CheXpert Plus and a novel multicenter dataset (CXR-27), for its capability to create CXR reports. The model is evaluated in a retrospective and in a prospective setting.

The authors have addressed my previous remaining feedback and suggestions (regarding unclear statements and suboptimal case selection) adequately. I have no further concerns at this time.

Reviewer #1 (Remarks on code availability):

I have not tried to run the code, but I read it and it has not changed since the last submission; as it largely builds on DeepSeeks repositories it is expected to be executable. The associated GitHub repository holds the code and instructions for inference (specifically, generating a report based on a provided CXR image). Training code is not provided, but will be made available after publication as per the author's comments.

Response: We sincerely thank you for your careful evaluation of our revised manuscript.

Reviewer #2 (Remarks to the Author):

I thank the authors for their thorough responses to my previous comments as well as comment from the other reviewers. The manuscript appears to have been strengthened on multiple fronts, including but not limited to the discussion section that now includes expanded discussion of the choice of models (generalist vs specialist). The updated supplementary appendices

including the expert panel training protocol and other minor updates to the clinical impact aspects of the manuscript appear to be well done. In my re-reading of the manuscript, I noted some of the study strengths including the emphasis on the prospective aspects as well as clinical impact. All in all, the authors addressed the comments and concerns I had. I have no further revision request.

Reviewer #2 (Remarks on code availability):

In my previous iteration of review, I have reviewed the code at a higher level including the readme and deemed it okay, although I did not compile the code myself to verify that it runs successfully.
Response: Thank you for your careful review and valuable comments. We are pleased that the revised manuscript now meets your expectations.

Reviewer #3 (Remarks to the Author):

The responses to my prior review comments appear to be appropriate with further discussion on tradeoffs between generalist vs. specific models, and better methodologic description of the human evaluation process.

I have no other major revision comments to add.
Response: We thank you for the time and expertise you devoted to reviewing our revised submission.

Reviewer #4 (Remarks to the Author):

Most of my major concerns have been mainly addressed, while remaining minor concerns have been already addressed by the other Reviewers.

Remaining notes:
Line 125: "significantly" should be "statistically significant".
Line 128: remove "notably"
Line 136-139: it's an unspecified subgroup analysis, P-values are not needed, only report confidence intervals.
Add "in the retrospective study" to the title of results section "Second outcomes" (line 140) (if it only reports results from retrospective analysis).

Response: We truly appreciate your recognition of our previous revisions. Thank you as well for identifying the remaining issues. We have revised the manuscript accordingly to ensure that these points are fully clarified.

Page 5, Lines 121-123: In terms of report quality, the AI-assisted group achieved a mean score of 4.36±0.50, statistically significantly higher than the standard care group's score of 4.12±0.80 (Mean of differences = 0.25, P < 0.001, 95% CI = 0.216-0.283) (Figure 3A).

Page 5, Lines 123-126: The agreement score of the AI-assisted group's reports, assessed using the RADPEER scoring system, was 4.30±0.57, ~~notably~~ higher than the standard care group's score of 4.14±0.84 (Mean of differences = 0.16, $P < 0.001$, 95% CI = 0.119-0.200) (Figure 3B).

Page 6, Lines 134-136: Subgroup analysis revealed that for complex cases (those with ≥ 3 imaging findings), the time advantage for the AI-assisted group remained substantial (165.1 ± 29.4 vs. 197.6 ± 26.9 seconds, Mean of differences = 32.5s, 16.4% reduction, ~~P < 0.001,~~ 95% CI = 31.6-33.4).

Page 6, Line 137: **Second outcomes in the retrospective study**
